# Effects of Dietary Fibers on Short-Chain Fatty Acids and Gut Microbiota Composition in Healthy Adults: A Systematic Review

**DOI:** 10.3390/nu14132559

**Published:** 2022-06-21

**Authors:** Valentina Vinelli, Paola Biscotti, Daniela Martini, Cristian Del Bo’, Mirko Marino, Tomás Meroño, Olga Nikoloudaki, Francesco Maria Calabrese, Silvia Turroni, Valentina Taverniti, Andrea Unión Caballero, Cristina Andrés-Lacueva, Marisa Porrini, Marco Gobbetti, Maria De Angelis, Patrizia Brigidi, Mariona Pinart, Katharina Nimptsch, Simone Guglielmetti, Patrizia Riso

**Affiliations:** 1 Department of Food, Environmental and Nutritional Sciences (DeFENS), Università Degli Studi di Milano, 20133 Milan, Italy; valentina.vinelli@unimi.it (V.V.); paola.biscotti@unimi.it (P.B.); daniela.martini@unimi.it (D.M.); cristian.delbo@unimi.it (C.D.B.); mirko.marino@unimi.it (M.M.); valentina.taverniti@unimi.it (V.T.); marisa.porrini@unimi.it (M.P.); simone.guglielmetti@unimi.it (S.G.); 2 Biomarkers and Nutrimetabolomics Laboratory, Department of Nutrition, Food Sciences and Gastronomy, Food Innovation Net (XIA), Nutrition and Food Safety Research Institute (INSA), Faculty of Pharmacy and Food Sciences, University of Barcelona, 08028 Barcelona, Spain; tomasmerono@ub.edu (T.M.); andrea.union@ub.edu (A.U.C.); candres@ub.edu (C.A.-L.); 3 Centro de Investigación Biomédica en Red de Fragilidad y Envejecimiento Saludable (CIBERFES), Instituto de Salud Carlos III, 28029 Madrid, Spain; 4 Faculty of Science and Technology, Free University of Bozen, 39100 Bolzano, Italy; olga.nikoloudaki@unibz.it (O.N.); marco.gobbetti@unibz.it (M.G.); 5 Department of Soil Plant and Food Sciences, University of Bari Aldo Moro, 70126 Bari, Italy; francesco.calabrese@uniba.it (F.M.C.); maria.deangelis@uniba.it (M.D.A.); 6 Unit of Microbiome Science and Biotechnology, Department of Pharmacy and Biotechnology, University of Bologna, 40126 Bologna, Italy; silvia.turroni@unibo.it; 7 Microbiomics Unit, Department of Medical and Surgical Sciences, University of Bologna, 40138 Bologna, Italy; patrizia.brigidi@unibo.it; 8 Max Delbrück Center for Molecular Medicine in the Helmholtz Association (MDC), 13125 Berlin, Germany; mariona.pinartgilberga@mdc-berlin.de (M.P.); katharina.nimptsch@mdc-berlin.de (K.N.)

**Keywords:** dietary fibers, short chain fatty acids, intervention studies, prebiotics, human gut microbiota

## Abstract

There is an increasing interest in investigating dietary strategies able to modulate the gut microbial ecosystem which, in turn, may play a key role in human health. Dietary fibers (DFs) are widely recognized as molecules with prebiotic effects. The main objective of this systematic review was to: (i) analyze the results available on the impact of DF intervention on short chain fatty acids (SCFAs) production; (ii) evaluate the interplay between the type of DF intervention, the gut microbiota composition and its metabolic activities, and any other health associated outcome evaluated in the host. To this aim, initially, a comprehensive database of literature on human intervention studies assessing the effect of confirmed and candidate prebiotics on the microbial ecosystem was developed. Subsequently, studies performed on DFs and analyzing at least the impact on SCFA levels were extracted from the database. A total of 44 studies from 42 manuscripts were selected for the analysis. Among the different types of fiber, inulin was the DF investigated the most (*n* = 11). Regarding the results obtained on the ability of fiber to modulate total SCFAs, seven studies reported a significant increase, while no significant changes were reported in five studies, depending on the analytical methodology used. A total of 26 studies did not show significant differences in individual SCFAs, while the others reported significant differences for one or more SCFAs. The effect of DF interventions on the SCFA profile seemed to be strictly dependent on the dose and the type and structure of DFs. Overall, these results underline that, although affecting microbiota composition and derived metabolites, DFs do not produce univocal significant increase in SCFA levels in apparently healthy adults. In this regard, several factors (i.e., related to the study protocols and analytical methods) have been identified that could have affected the results obtained in the studies evaluated. Future studies are needed to better elucidate the relationship between DFs and gut microbiota in terms of SCFA production and impact on health-related markers.

## 1. Introduction

Given the well-known role of gut microbiota on human health, it is not surprising that scientific interest in dietary strategies able to modulate its composition is rapidly increasing. In particular, the intake of dietary fibers (DFs) has recently received considerable attention for their direct and indirect beneficial effects on human health. DFs may act as prebiotics, a category of molecules that not long ago were redefined by the International Association for Probiotics and Prebiotics (ISAPP) as substrates that are “selectively utilized by host microorganisms conferring a health benefit” [1].

Because of their properties (e.g., resistance to digestion by host enzymes and fermentability by intestinal microorganisms), DFs can impact the gut microbial ecosystem, modifying its composition in terms of taxa presence/absence, relative abundance, and metabolism. Reportedly, different microbes possess differentiated capability in metabolizing DFs, resulting in the production of different end-products [2]. DFs have heterogeneous physicochemical features and their metabolization requires an impressive array of carbohydrate-active enzymes (CAZymes) differently encoded among gut microorganisms [3,4]. Hence, the fermentation of DFs by the gut microbiota is structure-dependent and may involve several biochemical pathways [3].

The major end-products deriving from the gut fermentative activity on DFs are short-chain fatty acids (SCFAs), specifically, acetate, propionate, and butyrate, together with gases, such as H_2_ and CO_2_ [5].

SCFAs produced by anaerobic gut bacteria have a positive influence in human health, such as maintenance of immune homeostasis, effect on metabolic processes, glucose homeostasis, intestinal barrier integrity, and appetite regulation [5,6]. Owing to their functional activities, these metabolites appear to have a beneficial role in the prevention and treatment of various diseases [7] and their levels, and those resulting from microbial producers in the gut, are in fact typically decreased in disease-associated dysbiotic contexts [8]. Therefore, the gut microbiota becomes the active link between DFs and the associated host health.

Although the crucial role of SCFAs on host gut and immune homeostasis is well known, the current literature regarding the role of DFs on human health focuses primarily on the effects on the gut microbiota, without considering these key metabolites. The finely tuned intestinal microbial ecosystem is characterized by functional redundancy, i.e., taxonomically different microorganisms may exert the same functions leading to the same metabolic products. Consequently, although the taxonomic structure of the intestinal microbial ecosystem may vary widely among people, the metabolic functions of the gut microbiota are substantially maintained in different subjects. Therefore, the assessment of the effect of a dietary intervention on the intestinal microbiome may be better assessed by focusing on specific activities or metabolites, such as the SCFAs, rather than just the gut microbiota taxonomic composition.

Based on these premises, the primary objective of this systematic review was to analyze the current literature in order to explore the results available on the relationship between DF intervention and SCFA production as a result of the modification of gut microbiota composition. In addition, we aimed to evaluate scientific evidence regarding the interplay between the type of DF intervention, the composition and the activity of the gut microbiota, and any other health-related outcome evaluated in the host.

## 2. Materials and Methods

The process of study selection was divided into two main steps:

(a) Development of a comprehensive database of human intervention studies investigating the role of confirmed and candidate prebiotics (Phase 1);
(b) Extraction of all the human intervention studies performed using DFs from the database described above (Phase 2).

### 2.1. Literature Search Strategy and Study Selection for the Database Development

In Phase 1, a systematic literature search was conducted using PubMed and Web of Science databases. The search was performed on the 20 January 2022. The following search terms were used: (dietary fiber OR food OR phenols OR bioactive OR phytochemical OR diet OR nutrient) AND (prebiotics OR microbiota OR fatty acids, volatile) AND (intervention study OR trial) AND (humans OR adult). To ensure completeness, the search was improved by screening the bibliographies of relevant review articles.

The search was set in the period between 1 January 2010 and 20 January 2022 following the steps reported in this section. This systematic review protocol was not registered.

Study selection was conducted by two independent reviewers (V.V. and P.B.) and disagreements were solved by consulting a third reviewer (M.M.).

Studies were selected if they investigated the effect of consuming prebiotics, both confirmed or candidate food/ingredients, on the modulation of SCFA profile, and/or gut microbiota composition in humans aged 18 years and over. To be included in a pivotal database, studies had to be human intervention trials of either acute (i.e., single-dose supplementation) or chronic prebiotic consumption and had to provide a characterization of the prebiotic content. Studies were considered eligible if they were published in English. Studies were excluded if prebiotics were combined with probiotics (i.e., synbiotics; because any beneficial effect could not be attributed specifically to the prebiotic under study). There were restrictions pertaining to age (<18 years within the target population), but not to other characteristics of study participants (e.g., BMI, health condition, and sex education).

### 2.2. Selection of Studies Related to Dietary Fiber Intervention

In Phase 2 of the present study, published literature specifically directed to the evaluation of the impact of DFs on SCFAs, and microbiota composition if available, was extracted from the database described above. For the analysis, only trials reporting the actual amount of tested fibers were included. In particular, the inclusion and exclusion criteria are reported below.

Inclusion criterion: Papers investigating the effect of foods rich in DFs or DF supplements on at least SCFAs in apparently healthy subjects.

Exclusion criteria: Papers that did not report any assessment of SCFA production; studies involving probiotics or synbiotics; studies with a target population with BMI > 30 kg/m^2^, age < 18 and >60 years; and studies with enteral formulas.

A more detailed list of eligibility criteria, developed by following the PICOS (Population, Intervention, Comparison, Outcome, Study design) format, is summarized in Table 1.

### 2.3. Data Extraction and Presentation and Quality Assessment

Data extraction from eligible studies was performed by 2 contributors (P.R., S.G.). A third author (V.T.) was involved in solving disagreements between authors during data extraction. The following data were extracted from each study: name of the first author and year; study population; study design; fiber intervention; control or placebo intervention; outcome variables; main findings on SCFAs, and, when available, microbiota composition and other outcomes. Trials evaluating both healthy and unhealthy groups were added only with data available on the healthy group.

The risk-of-bias tool was used for quality assessment using the following seven domains: (1) random sequence generation, (2) allocation concealment, (3) blinding of participants and personnel, (4) blinding of outcome assessment, (5) incomplete outcome data, (6) selective reporting, and (7) other bias. All domains were judged as high risk, unclear risk, or low risk. Any disagreement was resolved by consensus or seeking consultation with another author (D.M.).

Because of the expected heterogeneity, we were unable to conduct meta-analyses.

## 3. Results

### 3.1. Description of Selected Trials and Risk of Bias

**Figure 1:**
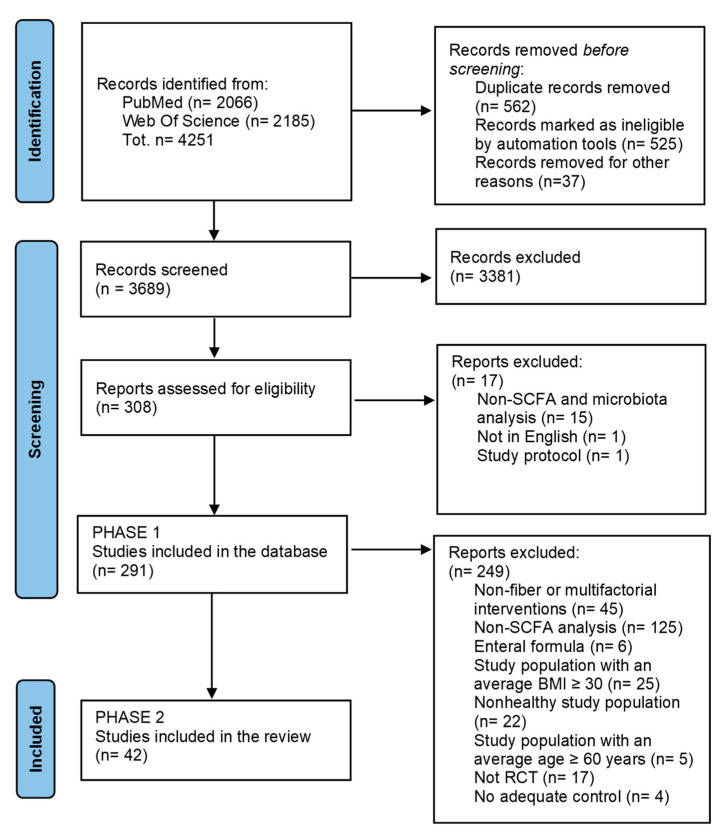
Flow diagram of studies included in the database (Phase 1) and in the systematic review (Phase 2) (PRISMA diagram).

As reported in the PRISMA diagram (Figure 1), in Phase 1 a total of 4251 records were identified from the PubMed and Web of Science database search, and no additional records were found by manual searching. After removing 562 duplicate articles, 3689 studies were screened and 3381 excluded based on title and abstract. After removing 17 studies, we developed a database consisting of 291 studies that were categorized based on BMI (BMI < 30 or BMI > 30), age (age < 60 years or age > 60 years), healthy status, prebiotic intervention (diet, food, or supplements), SCFAs, and/or gut microbiota modulation (Phase 1).

In Phase 2, to select studies focused on the effects of DF intervention on SCFA profile in healthy adults, 249 studies were excluded because they did not meet the inclusion criteria. At the end of the selection process, 42 RCTs were included in the qualitative analysis [9,10,11,12,13,14,15,16,17,18,19,20,21,22,23,24,25,26,27,28,29,30,31,32,33,34,35,36,37,38,39,40,41,42,43,44,45,46,47,48,49,50]. The main characteristics of the selected studies are reported in Tables S1–S3.

**Figure 2:**
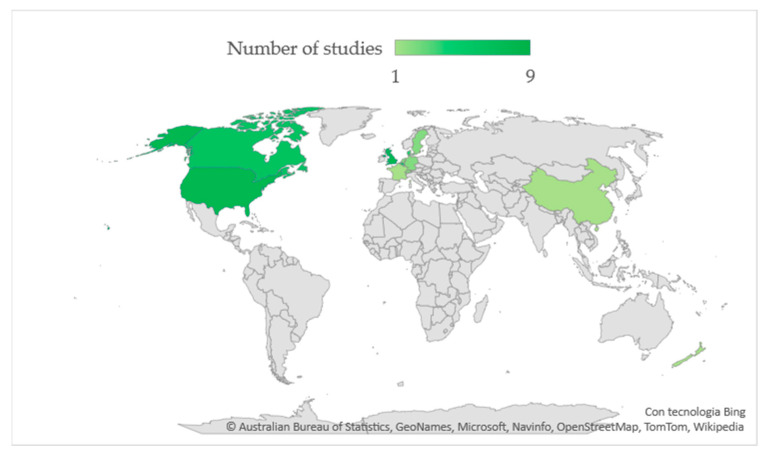
Countries where studies on dietary fibers were performed. The green indicates the number of studies performed in the different countries: the darker the green, the higher the number of studies conducted.

The number of studies from different countries is reported in Figure 2. As shown, USA and UK had the highest number of studies (*n* = 8), followed by Canada (*n* = 7), Belgium (*n* = 5), and Denmark (*n* = 4). Fewer studies were conducted in The Netherlands, Germany, Sweden, and Switzerland (*n* = 2); China, France, and New Zealand contributed with one study each.

Risks of bias within individual studies and across the studies are shown in Supplementary Figures S1 and S2, respectively. The results show the incomplete outcome of data (attrition bias) and the selective reporting (reporting bias) to represent the highest risks of bias.

#### 3.1.1. Study Design and Interventions Tested

The main characteristics of the 42 included studies are reported in Table S1. A total of 44 trials were available for detailed analysis from the 42 included studies since two of the publications [9,10] reported two separate trials. Specifically, 10 out of the 44 trials were parallel randomized controlled trials (RCTs), while 34 were cross-over RCTs. Four cross-over RCTs [11,12,13,14] did not include a washout period, which can be considered a limitation.

Most of the included trials (*n* = 35) evaluated the effects of supplementing specific DFs; most of them (*n* = 28) tested a single type of fiber, while 9 RCTs tested a fiber mix.

The most tested prebiotic fiber was inulin (IN) with 11 studies [10,14,15,16,17,18,19,20,21,22,23] assessing its effect on the SCFA profile. Two studies [15,20] evaluated the effects of an inulin-type fructan intervention on SCFAs; in one study [18], the effect of inulin supplementation with different wheat bran (WB) fractions was compared. Another study evaluated the prebiotic effect of xylo-oligosaccharide (XOS) in combination with inulin [22].

The other fibers used were: arabinogalactan [24], xylo-oligosaccharide (XOS) [25] IN + XOS [22], beta-glucans [26], digestion-resistant dextrin [27], extrinsic wheat fiber [9], resistant starch (RS) [28,29,30], AXOS + RS + glucans [31], galacto-oligosaccharides (GOS) [32], L-rhamonse (L-Rha) [17], polydextrose (PDX) [11,13,33,34], oligofructose (OF) [35,36], raffinose [12], soluble fiber [11,13], wheat bran extract (WBE) [36,37,38], 2′-fucosyllactose (FL), lacto-N-neotetraose (LNnT), 2′FL + LNnT [39], lupin kernel fiber, and citrus fiber [40].

Two studies [41,42] assessed the impact of eating bread with in situ produced arabinoxylan oligosaccharides (AXOS) obtained by xylanase treatment. The remaining trials tested the effects of a diet [43,44,45,46] or a specific food [12,47,48,49,50] providing a defined quantity of DFs.

Intervention doses of fiber supplementation ranged from 1.4 g/day [25] to 50 g/day [29], and treatment periods ranged from one meal (single dose) up to 3 months of regular intake with most treatments lasting 3 weeks [11,12,13,14,16,20,21,26,33,34,37,41,42,47].

Concerning total fiber intake registered during the interventions, data are reported in Table S2.

#### 3.1.2. Subject Characteristics

A total of 1377 subjects were involved in the 44 trials. The study population was mainly represented by healthy adults except for 156 subjects (in three studies) with mild hypercholesterolemia, moderate hypercholesterolemia, and overweight [40,46,48,49].

The target population had a normal BMI: the mean BMI ranged from a minimum of 20.9 kg/m^2^ [22,41] to a maximum of 27.9 kg/m^2^ [43]. Three studies reported different ranges in the inclusion criteria without specifying the average [16,44,49] while two studies did not report any data [12,28]. In one case, only the exclusion criteria about the BMI of population were reported [14], while in another the BMI median value was reported [41].

Only in two cases were the different effects of dietary fiber intervention evaluated by comparing healthy subjects and two unhealthy groups, i.e., hyperinsulinemic or overweight/obese. For these studies, we considered only the data obtained in the healthy target group.

The mean age of the subjects ranged from a minimum of 20 years [22] to a maximum of 55 years [45]. Three studies reported the age ranges of the subjects recruited, but not the group mean [14,23,49], while two studies reported the different age ranges in the inclusion criteria without specifying the mean [30,44]. One study reported the median age only [41].

Finally, two studies investigated the effects of DF intervention in adults and older subjects [28,32]; in this case, when presenting the results, we considered only the data obtained in the former.

### 3.2. Main Findings

#### 3.2.1. Analysis and Main Effects of Treatments on SCFAs

In the majority of cases, the samples used to analyze SCFA modulation were from feces (*n* = 40), except for three studies that used serum samples [17,19,30], three studies that used plasma samples [18,31,49,50], and one study using fecal water samples [44].

Concerning the techniques used for the evaluation of SCFAs, the most widely used was gas chromatography (GC) (22 trials); the remaining trials used: gas chromatography–mass spectrometry (GC–MS) [24,26,29,35,36,45] high-performance liquid chromatography (HPLC) [16,23,32,39,47,48], capillary gas–liquid chromatography (GLC) [15,22,25], gas chromatography–flame ionization detection (GC-FID) [40], liquid chromatography–tandem mass spectrometry (LC–MS/MS) [9], nuclear magnetic resonance (NMR) [34,44], ultraperformance liquid chromatography–tandem mass spectrometry (UPLC–MS/MS) [49], and ion exchange chromatography with conductivity detection [38]. Considering the different accuracy and reliability of the obtainable results, the technique used must be properly considered in the assessment of the data obtained.

First, it should be considered that colonic luminal SCFA concentrations, as well as fecal ones, are in the millimolar range and are relatively easy to quantify using GC [51]. However, SCFA concentrations in plasma and serum are considerably lower than in the lumen of the colon and range between 50 and 100 μmol/L for acetate and 0.5 and 10 μmol/L for propionate and butyrate [52]. Different methods to analyze SCFAs in biological samples involve: HPLC in combination with ultraviolet (UV) detection [53] or electrochemical detection (ECD) [54]; mass spectrometry (MS) [55,56] or gas chromatography (GC) with flame ionization detection (FID) [57] or MS [58]; capillary electrophoresis [59]. Of these methods, GC–MS is the most commonly used method for SCFA measurement in biological samples due to its higher sensitivity [60]. Among GC detectors, flame ionization detection (FID) is commonly used due to its inexpensive cost and operation, and its ability to detect a wide range of concentrations of organic compounds [61]. GC–FID is more reliable, reproducible, and sensitive for quantitative analysis than GC–MS, while GC–MS can provide more definite qualitative information and biomolecule identification [62]. The physicochemical properties of SCFAs, such as low vapor pressure and relatively high solubility in the aqueous phase, cause difficulties in the sample preparation [63]. Derivatization procedures are typically done in GC to enhance separations with increased resolution and response [64]. However, these procedures involve chemical reaction or concentration, which can lead to serious analyte loss due to the high volatility of SCFAs, together with risks of contamination and ghost peaks [65]. On the other hand, NMR methodology requires a very simple sample preparation step as compared to GC–MS and has higher reproducibility [66]. However, its sensitivity is lower and is not suitable for the measurement of SCFAs in serum or urine [67].

Finally, GC is preferred over HPLC for volatile compounds analysis, such as SCFAs, because: (1) GC usually shows higher sensitivity; (2) costs of equipment and analysis are lower; (3) chromatographic conditions are usually easier to set, considering that no mobile phase is used (instead, a carrier gas is used, but it does not participate in the chromatographic separation) and that a huge number of tailor-made stationary phases are available on the market. In addition, the use of hydrophilic interaction chromatography (HILIC) to determine SCFA may have advantages in terms of selectivity and specificity with respect to the common reversed-phase liquid chromatography (RFLC). However, HILIC is more difficult to setup and perform the analysis, and columns are usually expensive and prone to fast deterioration. Moreover, HPLC– or LC–MS/MS methods without chemical derivatization cannot distinguish between linear or branched SCFA isomers with the potential of yielding different concentrations due to co-quantification [68].

Last, the capillary gas–liquid chromatography (GLC) method is suitable for the routine separation of SCFA in intestinal fluids if the concentration of the acids to be determined amounts to more than 0.2 mmol L^−1^. However, GLC has a restricted sensitivity and the resolution of the packed column is an additional limitation [69].

Overall, based on the methodological considerations described above, no statistically significant differences in SCFAs were found following the dietary treatments when HPLC–MS/MS was used, regardless of the sample type (six studies used feces and one plasma). Most of the studies used GC-based methods, and those studies in plasma or serum samples more commonly found statistically significant changes in SCFAs (four studies out of six). In those studies analyzing fecal samples, almost half reported statistically significant results, while the other half could not demonstrate any effect. Last, only two studies used NMR to analyze SCFAs in fecal samples and only one showed statistically significant results, while the other did not find differences.

Regarding the total SCFAs, which correspond to the sum of acetate, propionate, and butyrate, seven studies reported a significant increase [15,22,31,37,40,41,45] while no significant changes were reported in five studies [12,29,36,43,46].

As for the individual SCFAs, a total of 26 studies showed no overall significant differences [9,10,12,14,16,17,18,19,20,21,23,24,25,27,28,32,33,34,35,36,38,39,43,47,48,49]. Two studies [40,42] reported a significant increase in acetate, butyrate, and propionate, while another two reported a significant decrease for all three SCFAs [11,13]. One study [29] showed that although DF consumption did not alter total SCFAs, different DF structures differentially affected individual SCFAs. Acetate significantly increased in seven studies [29,31,37,40,44,45,60] and significant decreased in two [15,22]. Butyrate significantly increased in eight studies [22,29,31,37,41,42,44,50] and decreased in one [46]. Finally, the DF intervention led to a significant increase in propionate in five studies [22,29,37,42,50].

The effects of DF supplementation on the SCFA profile were strictly dependent on dose [18,25,29,31,37] and on the type and structure of DFs [9,11,13,18,22,26,29,31,40,50]. In addition, few studies reported that the effects could be influenced by individual factors such as age [28] and gender [26].

Some of the studies also evaluated branched-chain fatty acids (BCFAs), with six showing no significant changes in the overall profile [21,30,33,36,38,48], while two reporting a significant BCFA reduction [11,13]. With specific regard to iso-butyrate, three studies [23,24,32] showed no significant differences, while one study [26] reported an increase only in women. The same study also observed a significant increase in iso-valerate and 2-methylbutyrate only in women. Two studies reported no significant changes in iso-valerate [23,36] while another three [24,42,44] reported a significant reduction. Finally, a significant decrease in valerate was reported by two studies [11,24] while one study [42] showed a significant increase. In addition to BCFAs, one study [13] reported that other fecal protein-based fermentative end-products (ammonia, phenol, indole) decreased after DF intervention. In other studies, DF consumption was shown to lead to decreased urinary excretion of phenol 20 and p-cresol [22,36,37,41]. Referring to specific DFs, inulin consumption has been associated with decreased ammonia levels [14], as is the case with PDX and soluble maize [11]. Consumption of PDX and soluble maize fiber was also associated with a decrease in 4-methylphenol and indole compared to the control group [11].

Other potential microbial metabolites were also assessed: lactate [23], succinate [32,34,38,44], enterolactone [26], formate [23,26], gamma-aminobutyric acid (GABA) [26], caproic [33], and hexanoic acid [24]. In particular, there was a significant reduction in succinate [44], enterolactone [26], hexanoic acid [24], and, only in men, 4-hydroxyphenylacetate, trimethylacetate, and dimethylacetate [44], while a significant increase was observed in GABA and formate only in men [26] and in lactate in the whole cohort [23]. In the other studies, no significant changes were reported for succinate [32,38], formate [23], or caproic acid [33].

#### 3.2.2. Analysis and Main Effects of Treatments on Gut Microbiota Composition

##### Overall Considerations

All but nine studies [17,18,19,26,31,36,40,44,50] used fecal material and DNA-based methods to determine the gut microbiota composition during the intervention period and at the end of post-intervention washout. Fourteen studies profiled the microbiota by 16S rRNA gene amplicon sequencing [9,10,13,20,21,24,28,29,30,35,38,39,45,46], eight with fluorescence in situ hybridization (FISH) [10,16,32,33,37,42,47,48], while the remaining trials used: quantitative PCR (qPCR) [11,12,15,22,23,27,32,33,49] culture, culture-based analysis [14], or a combination of diverse methods (e.g., culture-based analysis and 16S rRNA gene profiling, qPCR with 16S rRNA gene profiling, and terminal restriction fragment-length polymorphism (T-RFLP)) [12,25,32]. The exception was one paper [34] in which the authors used data from a previous study [33] to perform correlation analysis between bacterial groups and metabolites. The different methods applied are known to have their own biases and accuracy. The most common microbial groups evaluated by targeted gut microbiota analyses, apart from total bacteria, were *Bifidobacterium* spp., *Actinobacteria*, *Bacteroides* spp., *Lactobacillus* spp., *Enterococcus* spp. *and Clostridium* spp.

The gut microbiota composition varied between the DF intervention group and the control/placebo group, with three exceptions where no significant changes were observed [9,43,46]. In particular, studies with calculated diversity indexes reported that the within-sample (α-) microbial diversity decreased upon treatment [20,24,32,38], while β-diversity metrics varied based on DF intervention duration and diet type [32]. Nevertheless, most of the studies that calculated the diversity indexes (α- and β-) found no significant changes due to the DF intervention [9,10,13,21,25,29,30].

Regardless of the differences in treatment duration and detection methods employed, *Bifidobacterium* spp. increased in most studies [10,11,15,16,20,21,22,23,24,28,32,35,37,38,39,41,47,48,49]. The increase was sometimes abetted by the reduction in *Bacteroides* or/and *Clostridium* spp. [12,14,16,30] except for three studies where *Bacteroides* increased [11,24,27] and two studies where Clostridiaceae [13] and *Clostridium* spp. specific clusters [33] increased. As for lactobacilli, the results were mixed. An increase was reported in seven studies [14,16,22,38,42,49], a decrease in one [28] while their levels remained unaffected in four [11,23,25,28]. At the same time, for the phylum Firmicutes which includes *Enterococcus*, *Clostridium,* and *Lactobacillus* spp., an increase [20,24] but also a decrease [39] was reported. The results of several studies showed that the effects of DF supplementation on the gut microbial ecosystem were strictly dose-dependent [25,29,37].

### 3.3. Detailed Effects of DF Interventions on the Gut Microbial Ecosystem

In this section, the interaction between administered DFs and SCFA profile in healthy adults is analyzed in detail, by DF type. Almost all SCFA analyses were conducted using GC on fecal samples. When available, changes in the gut microbiota (and methods used) are also discussed.

#### 3.3.1. Arabinogalactan

The supplementation of 15 g/day of arabinogalactan for 6 weeks led to a decrease in isovaleric, valeric, and hexanoic acid, while no differences were found for SCFAs and isobutyrate compared to MD (as measured in feces using GC–MS). The trial was conducted on 30 healthy adults [24]. Regarding microbiota composition, α-diversity (Shannon and Simpson indices), was significantly lower with arabinogalactan intervention than with placebo at weeks 3 and 6 after adjustment for the respective baseline, while β-diversity measures were not significantly different between the two intervention groups and between weeks 3 and 6 and baseline. The Firmicutes/Bacteroidetes ratio was significantly lower in the treated group, due to a significant decrease in Firmicutes and a significant increase in Bacteroidetes. *Bifidobacterium* tended to increase with arabinogalactan supplementation compared to placebo. Other changes in potential SCFA producers from the Lachnospiraceae family were observed, including an increase in genera *Eisenbergiella* and *Howardella*. As expected, in the intervention group, the authors predicted (through PICRUSt) an overrepresentation of α-L-rhamnosidase, which is capable of releasing rhamnose from polysaccharides, including arabinogalactan proteins.

#### 3.3.2. Arabinoxylan Oligosaccharides (AXOS)

Two studies [41,42] analyzed how AXOS influenced fecal SCFA production. In the first, 27 healthy adults were enrolled to consume approx. 2.14 g/day of AXOS as 180 g of wheat/rye bread with AXOS for 3 weeks. As a result, total SCFAs, butyrate and, even if not significantly, acetate and propionate (determined through GC) increased from baseline [41]. As for the gut microbiota, a trend toward increased bifidobacterial proportions was found using FISH (*p* = 0.06). Interestingly, reduced urinary excretion of potentially harmful metabolites, such as phenol and p-cresol, was also observed. An almost equivalent amount of AXOS (2.2 g/day), produced in situ with endoxylanase-treated breads, consumed for 3 weeks by 40 healthy adults, resulted in a significant increase in most SCFAs as determined by GC: butyrate and propionate compared to pre-AXOS; and valerate compared to placebo, control, and baseline [42]. Combined acetate, propionate, and butyrate also increased compared to pre-AXOS, while iso-valerate decreased, despite not significantly. The gut microbiota was profiled by FISH; some compositional changes were observed but not exclusive to the AXOS intervention group.

#### 3.3.3. Galacto-Oligosaccharides (GOS)

Only one of the selected studies evaluated the effects of GOS on fecal SCFAs by HPLC [32]. Twenty-four healthy adults consumed 21.6 g/day Biotis™ GOS (containing 15.0 GOS) for 4 weeks; no significant changes in SCFAs, isobutyrate, and succinate were recorded, either from baseline or compared to MD (placebo). Based on 16S rRNA gene profiling, bacterial diversity (InvSimpson) significantly decreased after four-week GOS supplementation. Similarly, β-diversity metrics (weighted UniFrac) showed significant changes in gut microbiota structure after 4 weeks of treatment. Taxonomically, the authors found a significant increase in bifidobacteria, which was confirmed by qPCR.

#### 3.3.4. Inulin (IN)

One study aimed to examine the effect of consuming 7 g/day (trial 1) or 3 g/day (trial 2) of inulin for 4 weeks on 25 healthy adults; in neither trial were there significant changes in fecal SCFAs, as determined by GC [10].

Using the same technique, another study confirmed that the fecal SCFA and BCFA profile of 29 healthy adults remained unchanged after consumption of both 5 and 7.5 g/day of agave inulin for 3 weeks [21]. However, a positive association was observed between total dietary fiber intake and fecal butyrate.

Similarly, administration of 20 g/day of inulin for 3 weeks to 12 healthy male adults [14] and for 4 weeks to 32 women with low-iron status [23] had no significant effect on the fecal profile of SCFAs (analyzed by GC and HPLC, respectively). However, a controlled diet with 20 g/day of chicory IN into low-fat vanilla ice cream led to a significant increase in the acetate/propionate ratio compared to the control [14]. As for the second study, it should be noted that an increase in lactate was observed compared to baseline and control, which correlated significantly with propionate [23].

As expected, even acute administration of 22.4 g of inulin to 13 healthy adults [17] and 24 g of inulin to 9 adults [19] did not induce significant changes in SCFA levels, as measured in serum by GC. However, in the second study, a significant time × treatment interaction for acetate was observed.

The effect of very-long-chain inulin (VLCI) supplementation was also tested in 32 healthy adults [16]. Consumption of 10 g/day for 3 weeks, compared to 10 g/day of MD, again did not result in significant changes in the fecal profile of SCFAs, as measured by HPLC.

Despite this, studies that have profiled the gut microbiota or at least some bacterial groups have shown some noteworthy compositional changes. In particular, all but one [14] were consistent in highlighting an increase in bifidobacteria, albeit with different techniques (16S rRNA gene amplicon sequencing [10,21], qPCR [23], and FISH [16]), confirming the well-known bifidogenic effect of inulin. Furthermore, the intervention with VLCI [16] resulted in an increase in *Atopobium* and *Lactobacillus*–*Enterococcus*, and a decrease in *Bacteroides*–*Prevotella*. Agave inulin supplementation at 7.5 g/day [21] significantly reduced the relative abundance of the sulfidogenic pathobiont *Desulfovibrio* (which correlated negatively with total fiber intake and positively with fecal 4-methylphenol, a marker of proteolytic fermentation), along with *Lachnobacterium* and *Ruminococcus*. In the study where inulin was administered at 7 g/day [10], *Lachnospira* and *Oscillospira* decreased, while *Nesterenkonia, Brevibacterium,* and *Cellulomonas* increased significantly, probably due to the small amount of other fiber in the isocaloric snack bars, given the cellulose-degrading potential of the latter. Finally, supplementation of 20 g/day of chicory inulin increased total anaerobes and *Lactobacillus* spp., and reduced enterobacteria, along with ammonia levels [14].

#### 3.3.5. Lupin Kernel Fiber (LF) and Citrus Fiber (CF)

In 52 moderately hypercholesterolemic adults, consumption of a high-fiber diet containing 25 g/day of lupin kernel fiber (LF) or citrus fiber (CF) was tested [40]. LF supplementation was associated with a significant increase in total and individual SCFAs in feces (as assessed by GC–FID) compared to baseline and control. On the other hand, CF supplementation led to a significant increase in total SCFAs from baseline, and acetate compared to baseline and control. Interestingly, LF and CF also resulted in increased primary bile acid excretion and reduced secondary bile acid excretion, respectively. The gut microbiota composition was not determined.

#### 3.3.6. L-Rhamnose

One RCT in which healthy unrestrained eaters consumed 25.5 g/day of L-rhamnose alongside a standardized breakfast and lunch, found that this acute ingestion did not lead to any significant postprandial serum changes in propionate, acetate, and butyrate, as based on GC determinations [17]. After each meal, blood was sampled every 15 min for the first hour, then half-hourly. The gut microbiota composition was not assessed.

#### 3.3.7. Oligofructose (OF)

In 19 healthy adults, the consumption of a low FODMAP diet supplemented with 14 g/day of OF for 1 week did not change fecal SCFAs (assessed by GC-MS) compared to baseline and control (18 healthy adults consuming a low FODMAP diet supplemented with MD) [35]. On the other hand, the gut microbiota was affected, particularly with an increase in *Bifidobacterium* and a decrease in Lachnospiraceae. In addition, 21 taxa were found to be significantly associated with SCFAs, including Lachnospiraceae and *Roseburia*, which correlated positively with butyrate and acetate, and *Anaerostipes*, which showed a negative association with isobutyrate and valeric acid.

In another study in 19 healthy adults, the supplementation of a habitual diet with 15 g/day (during the first week) and then 30 g/day (during the second week) of OF again did not affect total and individual SCFAs in feces (as assessed by GC–MS), compared to baseline and placebo [36]. The gut microbiota was not assessed.

#### 3.3.8. O-Fucosyllactose (2′FL) and/or Lacto-N-Neotetraose (LNnT) and Their Mix

In an HMO-supplementation study, consumption of 2′FL and/or LNnT at various daily doses (5, 10, or 20 g) and mixes was tested for 2 weeks in 90 healthy adults [39]. No significant changes in fecal levels of acetate, butyrate. or propionate were observed in any case (by HPLC). Compared to baseline, LNnT consumption showed an increase in Actinobacteria at all doses and decreases in Firmicutes at 20 g and Proteobacteria at 10 g. An increase in Actinobacteria was also shown by 2′FL at 5 and 10 g and the mix (2:1 mass ratio) at 10 and 20 g. The mix also resulted in a decrease in Firmicutes at 20 g. Finally, both 2′FL and LNnT led to an increase in bifidobacteria. Gut microbiota data were obtained through 16S rRNA gene amplicon sequencing.

#### 3.3.9. Polydextrose (PDX)

Two different studies analyzed the effect of 8 g/day of PDX for 3 weeks and both found no significant changes in any of the fecal metabolites, including SCFAs, BCFAs, dimethylamine, succinate, phenylacetate, and n-caproid acid, evaluated by NMR or GC, compared to the control and/or baseline [33,34]. The first study enrolled 31 healthy adults [33]; the second enrolled 12 healthy adults [34]. In the first, FISH and qPCR analysis revealed many changes in gut microbiota composition, including an increase in beneficial, SCFA-producing groups such as *Clostridium* cluster IV [33]. In the second, associations between PDX and acetate and propionate were identified, along with a strong correlation between fecal metabolites and bifidobacterial amounts and another correlation between *Bacteroides* and acetate, based on previously collected microbiome data [34].

Other researchers studied the effects of 21 g/day of PDX, compared to the consumption of soluble maize fiber, for 3 weeks on healthy adult men in two different trials [11,13]. All subjects (21 men in the first study) [11] and 20 men in the second [13] received three snack bars/day. Based on GC determinations, acetate, propionate, and butyrate decreased with PDX compared to soluble maize fiber, soluble corn fiber, and no supplemental fiber (NFC), as well as fecal protein-based fermentative end-products (e.g., ammonia, phenol, indole, and total BCFAs) [11,13]. In the first trial [11], soluble maize fiber led to increased levels of bifidobacteria, as measured by qPCR, while in the second [13], 16S rRNA gene profiling showed a number of potentially beneficial shifts in the gut microbiota driven by PDX, including greater proportions of Clostridiaceae and Veillonellaceae.

#### 3.3.10. Raffinose

After supplementation with 5 g/day of raffinose for 3 weeks to 12 healthy adults [12], the level of total and individual SCFAs in feces (determined by GC) remained unchanged. Based on T-RFLP and qPCR, a decrease in *Clostridium* clusters I/II and XI, including pathogenic and putrefactive bacteria, was reported.

#### 3.3.11. Resistant Dextrin

In a group of 48 healthy adults, administration of 10, 15, or 20 g/day of resistant dextrin for 2 weeks was examined [27]. The results revealed no significant changes in fecal SCFA production from baseline, as determined by GC. Based on qPCR, the amounts of *Bacteroides* increased significantly while those of potentially harmful *Clostridium perfringens* decreased from baseline. Furthermore, an increase in β-glucosidase activity was found, suggesting adaptation of the gut microbiota to the new dietary substrates, possibly optimizing energy harvesting.

#### 3.3.12. Resistant Starch (RS)

In a dose–response trial, 40 healthy adults consumed three structurally distinct type-IV resistant starches (RS4s) to achieve a gradual increase in maize, potato, or tapioca RS4 to 10, 20, 35, and 50 g/day [29]. In general, RS4 supplementation did not change the total SCFA concentration in feces (as measured by GC–MS); however, different RS4s showed distinct effects on individual SCFAs. In fact, compared to baseline, maize RS4 significantly increased the concentration of butyrate and its relative proportions; a reduction in the relative proportion of propionate and in the BCFAs to SCFA ratio was also observed. At the dose of 35 g/day, tapioca RS4 led to a significant increase in propionate from baseline; such an increase was also significantly higher than for maize RS4. Similarly, to maize RS4, consumption of tapioca RS4 at 35 g/day was associated with a reduction in the BCFAs to SCFA ratio, compared to both potato RS4 and placebo. Interestingly, in this trial, a dose–response relationship with a plateau at 35 g was also observed. As for the gut microbiota (profiled through 16S rRNA gene amplicon sequencing), higher doses of maize and tapioca RS4s resulted in a decrease in α-diversity, especially evenness. The effects on the composition were instead distinct and almost completely substrate specific. In particular, maize RS4 resulted in the enrichment of the butyrate producer *E. rectale*, together with *Oscillibacter* spp., *Ruminococcus* spp., and *Anaeromassilibacillus* spp., while tapioca RS4 in the enrichment of *P. distasonis*, together with *F. prausnitzii* and *Eisenbergiella* spp. Confirming the close associations between compositional and functional changes, Spearman’s correlation analysis showed that maize RS4-induced shifts in butyrate proportions were positively correlated with the increase in *E. rectale* (rs = 0.41, q = 0.07), while tapioca-RS4-induced shifts in propionate proportions were positively correlated with the increase in *P. distasonis* (OTU21; rs = 0.49, q = 0.03).

In another study, the administration of 40 g of high-amylose RS2 for 4 weeks to 19 adults led to a significant increase in serum acetate (as determined by GC) compared to control [30]. In addition, a positive correlation was found between changes in acetate and postprandial change in GLP-1 at 30 min. Gut microbiota profiling through 16S rRNA gene amplicon sequencing revealed many changes, including a decrease in *Anaerostipes*, *Bacteroides*, *Blautia*, *Holdemanella*, *Coprococcus_1*, *Coprococcus_3*, *Lachnoclostridium*, *Lachnospiraceae_UCG-004*, *Erysipelotrichaceae_UCG-003*, *Paraprevotella*, *Phascolarctobacterium*, *Ruminiclostridium_6*, *Ruminococcaceae_UCG-002*, and *Eubacterium_eligens*_group, and an increase in *Ruminococcaceae_UCG-005*. According to the authors, these changes were generally consistent with the available literature and could help explain the beneficial effects of RS.

Finally, a study in 42 mid-age adults revealed that the consumption of 30 g/day of MSPrebiotic® RS (unmodified potato RS) for 3 months did not lead to any significant changes in fecal SCFAs, as assessed by GC [28]. As for variations in the compositional structure of the gut microbiota (profiled through 16S rRNA gene amplicon sequencing), the authors found a decrease in α-diversity, an increase in the relative abundance of *Bifidobacterium* (particularly *B. ruminantium*) and *R. bromii* (a key player in the initial RS breakdown, which could aid subsequent degradation by bifidobacteria), while a reduction in the mucus-degrading species *R. torques* was reported.

#### 3.3.13. Xylo-Oligosaccharide (XOS)

Consuming 1.4 or 2.8 g/day of XOS for 8 weeks did not significantly change fecal SCFAs (measured by GLC) compared to baseline and placebo [25]. Results were different when healthy young adults consumed 5 g/day of XOS or 3 g inulin + 1 g XOS for 4 weeks: both XOS and INU-XOS led to an increase in butyrate, propionate, and propionate/acetate ratio, and a decrease in acetate, compared to the placebo group (MD); from baseline, the two dietary interventions significantly changed the levels of all three individual SCFAs. In addition, INU-XOS promoted the rise in total SCFA levels compared to MD [22].

In both studies [22,25], *Bifidobacterium* levels, although measured using different techniques (culture-based in the former and qPCR in the latter), were higher after treatment compared to placebo. Moreover, high-dose XOS resulted in increased proportions of the butyrate producer *Faecalibacterium* and *Akkermansia* (as determined by 16S rRNA gene profiling), while the INU-XOS combination led to a moderate increase in lactobacilli [22]. Notably, the authors also found a XOS-related increase in α-glucosidase and β-glucuronidase activities, suggested as possible positive markers of butyrogenic strains [22]. 

#### 3.3.14. Wheat Bran Extract (WBE)

As for WBE, one study reported the absence of significant changes in total and individual SCFAs in feces (as assessed by GC–MS) after supplementation at 15 g/day (week 1) and 30 g/day (week 2) to 19 healthy adults, compared to baseline and placebo [36]. However, it should be noticed that isovalerate and p-cresol significantly decreased from baseline. The gut microbiota composition was not determined.

Another study evaluated two different doses of 3 and 10 g/day of wheat bran extract (WBE) containing AXOS (2.4 and 8 g/day, respectively), consumed for 3 weeks by 57 healthy adults [37]. Based on GC determinations, total SCFAs, acetate, and propionate significantly increased, as well as butyrate at the higher dose compared to placebo; propionate also increased with 3 g/day WBE compared to placebo. As for the gut microbiota, FISH analysis showed an increase in bifidobacteria at the higher dose compared to placebo. Notably, urinary p-cresol excretion was significantly decreased at 10 g/day WBE, indicating reduced protein fermentation.

Finally, in 24 healthy adults, a high dose (15 g/day) of AXOS resulted in no changes in SCFAs, BCFAs, succinate, and lactate compared to MD. There was a decrease in acetate and butyrate from baseline but not significant. It should be noted that, unlike previous studies, SCFAs, BCFAs, succinate, and lactate were measured by ion exchange chromatography with conductivity detection [38]. Based on 16S rRNA gene amplicon sequencing, the α-diverstity of the gut microbiota was significantly reduced and there were some compositional changes, including especially an increase in bifidobacteria.

#### 3.3.15. Fiber Mix

In six studies [9,15,18,20,26,31] the effect of a dietary intervention with a fiber mix on the fecal and plasmatic SCFA profile was evaluated. A prominent feature in most of these studies was the presence of inulin.

Nineteen healthy adults were randomly assigned to the following 4 arms: (1) AXOS: 8.9 g/portion; glucans: 1.5 g/portion; RS: 6.6 g/portion (WWB + AXOS + RS); (2) AXOS: 18.4 g/portion; glucans: 3.1 g/portion; RS: 1.0 g/portion (WWB + hiAXOS); (3) AXOS: 0 g/portion; glucans: <0.15 g/portion; RS: 15 g/portion (WWB + hiRS); (4) AXOS: 0 g/portion; glucans: <0.15 g/portion; RS: 1.2 g/portion (WWB, control) [31]. An increase in all plasma markers evaluated (by GC) was reported: total SCFAs and acetate with AXOS compared to WWB; butyrate with WWB + hiAXOS vs. WWB; acetate, butyrate, and total SCFAs at fasting and during the postprandial period with increasing content of AXOS; total SCFAs at fasting, which were significantly related to a decrease in glucose response in the later postprandial phase; and breath H_2_, which was strongly correlated with total SCFAs during the experimental day. No microbiota data were provided.

After recruiting 20 adults, another study conducted two trials for 5 days, one with food products (trial 1) and the other with drinks (trial 2) both enriched with 10 g of dietary fiber concentrate extracted from the wheat plant (66% cellulose, 28% hemicellulose) [9]. No significant changes were found in fecal SCFAs (as assessed by LC–MS/MS), except for an increase in acetate with fiber-enriched foods (trial 1) compared to fiber-enriched drinks (trial 2). The gut microbiota was profiled through 16S rRNA gene amplicon sequencing, but no alterations were found.

In a study evaluating 30 healthy adults consumption of 15 g/day of β2-1 fructan for 4 weeks (50:50 mixture of inulin and short-chain oligosaccharides) significantly increased the total SCFA concentration, specifically increasing the proportion of propionate and butyrate while decreasing that of acetate, compared to MD. In addition, except for heptanoic acid, reduced proportions of BCFAs were observed. SCFA and BCFA concentrations were determined in feces by GLC [15]. Regarding the gut microbiota, qPCR analysis showed a significant increase in *Bifidobacterium* spp. Another study evaluated consumption of 16 g/day of inulin-type fructan (50:50 inulin to fructo-oligosaccharide mix) for 3 weeks in 14 participants who routinely consumed low dietary fiber (LDF;) and 20 participants who regularly consumed high dietary fiber (HDF;) [20]. No significant changes were found in fecal SCFAs compared to baseline, as assessed by GC. The gut microbiota profiling (through 16S rRNA amplicon sequencing) showed a reduction in α-diversity in the HDF group while an increase in the LDF group, and several compositional changes, including an overall increase in bifidobacteria regardless of the habitual fiber intake. Furthermore, in the HDF group, the authors observed an increase in *Faecalibacterium* but a decrease in other SCFA producers, such as *Coprococcus*, *Dorea*, and *Ruminococcus*.

Consumption of a standard breakfast supplemented with 10 g ^13^C-inulin and 20 g of a WB fraction (unmodified WB vs. WB with a reduced particle size (WB RPS) vs. destarched pericarp-enriched WB (PE WB)) did not change the cumulative plasma concentrations of ^13^C-SCFAs (as assessed by GC), nor the relative proportions of acetate, propionate, and butyrate, when compared to control [18]. Dietary fiber intervention was evaluated in 10 healthy adults. The gut microbiota was not profiled.

Finally, in a study to evaluate the effect of consuming 3 g/day of beta-glucans from different sources for 3 weeks, gender-specific variations in fecal SCFA levels (determined by GC-MS) were observed [26]. The study was conducted in 14 healthy adults, including eight males and six females. Notably, in the male group, supplementation increased total SCFAs and formate with barley beta-glucans compared to those of oat, and gamma-amino-butyrate and palmitoleic acid with mutant barley compared to control. In the female group, the authors observed an increase in isobutyrate, isovalerate, and 2-methylbutyrate with barley beta-glucans compared to oat beta-glucans, and a decrease in enterolactone with mutant barley compared to control. Again, the gut microbiota was not profiled.

#### 3.3.16. Specific Foods and Diets

In the other selected studies, food and diets with a specific amount of dietary fiber were tested. For example, the consumption of 200 g/day of canned chickpeas (corresponding to about 3–5 g of oligosaccharides) for 3 weeks in 12 healthy adults did not result in significant changes in fecal levels of total or individual SCFAs, as assessed by GC [12]. As for the gut microbiota, R-TFLP and qPCR analysis showed a reduction in *Clostridium histolyticum*–*Clostridum lituseburense* groups (including pathogenic and putrefactive taxa), and ammonia-producing bacteria.

No significant changes in fecal SCFA levels (determined by UPLC–MS/MS) were observed even after supplementation with 40 g/day of a commercially available oat-based product (containing 1.4 g of beta-glucans) for 6 weeks to 34 healthy adults with mild hypercholesterolemia) [49]. On the other hand, qPCR analysis showed an increase in lactobacilli and bifidobacteria (although the latter was not significant). The absence of changes in fecal SCFAs was confirmed by HPLC after the administration of 48 g/day of maize-derived whole-grain cereal (total fiber 14.2 g) for 3 weeks in 32 healthy adults [47], and after consumption of 45 g/day of whole-grain oat granola (WGO) for 6 weeks by 30 mildly hypercholesterolemic or glucose intolerant adults [48]. In both studies, an increase in bifidobacteria was found using FISH, along with an increase in lactobacilli and total bacteria with WGO.

Conversely, some changes in plasma SCFA levels were determined by GC in a study testing in 17 healthy adults) different evening meals in which the main proportion of carbohydrates was derived from: (1) ordinary barley kernels (OB); (2) OB cut 1–2 times (cutOB); (3) barley kernels with elevated amounts of amylose (HAB); (4) barley kernels with elevated amounts of β-glucans (HBB); (5) WWB added with high-RS corn starch (WWB + RS); (6) WWB with added similar amounts of RS and DF from barley (WWB + RS + DF); and (7) one-half portion of OB bread (1/2 OB) [50]. Specifically, HAB and HBB significantly increased butyrate compared to WWB. In addition, a positive correlation between butyrate and indigestible carbohydrates was found, and between butyrate and breath H_2_. On the other hand, butyrate at fasting was inversely related to serum insulin response and an inverse association between butyrate and acetate and postprandial glucose response was found.

The latest selected studies aimed to examine the effect of a diet rich in whole grains (WG) on the SCFA profile. One of these showed that the consumption of a WG-rich diet (total fiber: 32 g/day) by 17 healthy adults led to an increase in acetate and butyrate and a decrease in isovalerate compared to a refined grain (RG)-based diet; in addition, women experienced a reduction in succinate levels [44]. In this study, the metabolic profile was analyzed in fecal waters by NMR. Similarly, a WG diet (40 ± 5.0 g fiber) consumed for 6 weeks by 41 healthy adults (was compared with a RG diet (21 ± 3 g fiber) consumed for 6 weeks by 40 healthy adults [45]. The WG diet resulted in an increase in fecal acetate and total SCFAs compared to RG, as assessed by GC–MS. This study also profiled the gut microbiota through 16S rRNA amplicon sequencing, showing some favorable changes, namely an increase in *Lachnospira* and *Roseburia* (although the latter was just a trend) and a decrease in enterobacteria. In contrast, no changes were found in fecal levels of total and individual SCFAs and BCFAs (as determined by GC) when 33 healthy adults (consumed diets high in WG (>80 g/day) for 6 weeks [43]. The authors also analyzed some groups of the gut microbiota by FISH but did not find any compositional changes. Finally, a study investigated the effects on fecal SCFAs (by GC) of consuming whole-grain wheat (WGW; total dietary fiber, 34.9 ± 11.9 g) and whole-grain rye (WGR; total dietary fiber, 31.1 ± 10.9 g) compared to refined cereals (RW; total dietary fiber, 22.9 ± 9.2 g), in 70 overweight, otherwise healthy adults [46]. No significant changes in total SCFAs, acetate, and propionate were found with WGW or WGR from baseline. Butyrate decreased with RW compared to the two dietary interventions. Associations between plasma alkylresorcinols and total SCFAs, butyrate, and acetate were confirmed by dose–response analyses. Again, no changes were shown in the gut microbiota (profiled through 16S rRNA gene amplicon sequencing).

#### 3.3.17. Total Fiber Intake during Specific DF Interventions

In addition to the type of fiber, an additional source of heterogeneity among studies was related to the dose of fiber used in the different interventions. As shown in Table S2, almost all trials, except three, reported the amounts of fiber provided by the tested DF or food or diet. The daily doses largely varied among studies. For instance, among studies testing inulin, the doses used were 5 and 7.5 g [20] but also 20 g [23] to assess the impact of agave IN and the same amount in a trial investigating the impact of chicory IN [14], reaching 24 g in another [19].

While fiber provided by the tested foods was generally reported in most of the studies, daily total fiber intake during the interventions was estimated in only a few trials. However, also among studies providing this information, a large variability was observed. For instance, a daily total fiber consumption of about 19 g/day, thus lower than dietary recommendations, was provided in the study by Walton and colleagues [42], and by Connolly et al. who tested the effect of 2.8 g fiber and 1.3 g β-glucan (from a 45 g/day serving of product) [48]. On the other hand, the highest daily intake was estimated in the study by Zhang et al. [30] who investigated the impact of 40 g/day high-amylose RS2 in an intervention arm providing about 55 g of fiber, and in the study by Fechner and coworkers [40] in which 25 g/day lupin kernel fiber from experimental foods were included in a dietary pattern providing a total of 59 g DF, thus much higher than the suggested dietary target.

### 3.4. Link between 16S rRNA Gene Amplicon Sequencing Data and SCFA Levels

In order to infer a link between bacterial taxa and SCFA metabolism, in this section we report conclusive results based only on those studies where the gut microbiota composition relied on 16S rRNA gene amplicon sequencing, i.e., where the whole bacterial community was characterized rather than a few selected taxa. As previously mentioned, fourteen studies analyzed the microbiota using this untargeted method. Below we voluntarily avoid commenting on the target region used for the amplification step, the quality of the denoised reads, and the heterogeneity of the population cohorts, but we describe the differentially represented taxa and their association with SCFAs, as detected in each of the DF interventions.

The influence of diet on gut health, including the immune system, is firstly evidenced by those dietary regimens rich in whole grains (WG) [45,46] that add the beneficial effects of vitamins, trace elements, fibers, and many other potential effectors. When a WG diet was compared with a refined grain (RG) diet [45], an increase in the SCFA producer *Lachnospira* and a decrease in proinflammatory Enterobacteriaceae were detected, along with changes in stool acetate and total SCFAs. Moreover, a positive correlation emerged between *Lachnospira* and *Roseburia* and acetate and butyrate, respectively.

In a study based on the administration of inulin-type fructans [20], although several microbial changes were detected including an increase in the relative abundance of *Bifidobacterium* and a decrease in genera *Coprococcus, Dorea, Ruminococcus*, and *Oscillospira*, no difference in SCFA concentration was found after dietary treatment. The increase in the genus *Bifidobacterium* was confirmed in another cross-over study where the dietary intervention was based on the same type of fiber. Again, the measurement of SCFAs did not show any significant difference probably due to their absorption in the colon [14]. Agave inulin fiber supplementation [21] also did not lead to changes in SCFA levels but increased the proportions of *Bifidobacterium*; furthermore, a negative correlation was found between total fiber intake and the bacterial genus *Desulfovibrio*.

Treatments with resistant starches (RSs) have also been shown to influence the fecal bacterial metacommunity and consequently the metabolism of SCFAs. In particular, RS dietary intervention [30] promoted an increase both in various genera (Ruminococcaceae_UCG-005, *Akkermansia*, *Ruminococcus*_2, *Victivallis*, and *Comamonas*) and in the serum level of acetate. About discrete RS structures, crystalline maize RS4 and cross-linked tapioca RS4 led to increased relative abundances of *Eubacterium rectale* and *Parabacteroides distasonis*, respectively, which directed SCFA output toward either butyrate or propionate [29].

The administration of an arabinogalactan product obtained from larch extraction [24] did not lead to a significant change in acetate, propionate, butyrate, and isobutyrate, while the other BCFAs decreased. At the genus level, *Bifidobacterium*, one of the taxa responsible for lactate and acetate metabolism, showed an increasing trend in treated versus placebo subjects. Similarly, acute and long intake of the wheat bran extract arabinoxylan–oligosaccharide (AXOS) led to an increase in *Bifidobacterium* over time but no differences in fecal and plasma SCFA levels were found between the AXOS and the placebo group [38]. Furthermore, the supplementation of a DF concentrate extracted from the wheat plant [9] and composed mostly of cellulose and hemicellulose, did not result in significant differences in fecal SCFAs when compared to a control diet, but led to increased relative abundances of *Blautia faecis* (Lachnospiraceae family).

A strong correlation between total fecal SCFAs and Lachnospiraceae was also found in subjects receiving polidextrose (PDX) and corn fiber (SCF) compared to a control diet [13], supporting that this family is a major producer of SCFAs. The same study highlighted the increase in one of the major butyrate producers, i.e., *Roseburia*. On the other hand, the PDX-enriched diet led to a decrease in Lachnospiraceae and Eubacteriaceae. *Faecalibacterium prausnitzii*, a butyrate-producing species, together with *Roseburia*, *Phascolarctobacterium,* and *Dialister,* were increased in both PDX and SCF administered subjects, whereas *Lactobacillus* was greater only after SCF intake. Fecal acetate, propionate, and butyrate concentrations were lower in PDX versus SCF diet.

Finally, a diet low in fermentable, oligo-, di-, mono-saccharides, and polyols (FODMAPs) [35], in addition to either maltodextrin (MD) or oligo-fructose (OF), led to a reduction in bifidobacteria and SCFA-producing clostridial species and an increase in *Ruminococcus torques*, a mucus-degrader bacterium typically associated with irritable bowel syndrome (IBS) [70].

### 3.5. Other Health-Related Findings Considered in Selected Studies

In this section, direct and indirect effects on health-related parameters are discussed (Table S3).

*Body composition*. Regarding body weight: two studies reported no changes [10,21], one study a trend toward an increase in RG compared to WG [43], another one a decrease, as well as in BMI and waist circumference following CF and LF treatment vs. baseline [40]; no significant changes were registered in anthropometric measurements compared to the control group [30,47] or from baseline [9], or both (i.e., baseline and control) [43].

*GI symptoms*. Twenty studies analyzed tolerance in terms of gas/flatulence, nausea, vomiting, abdominal cramping, abdominal distention/bloating, borborygmus/stomach rumbling, burping, and reflux [11,14,15,16,17,20,21,22,24,27,28,29,30,31,32,33,37,38,39,46]. Nine studies did not find differences in GI symptoms such as flatulence and abdominal pain [16,24,28,30,31,32,33,39,46] and gastric emptying [38]. On the other hand, some studies showed an increase in moderate flatulence [11,20,22,27,29,37,46], mild and moderate bloating [16,22,39], indigestion [15], abdominal pain [15,27], and frequency of self-reported adverse GI events [15], reflux [11], rumbling [39], and GI symptoms in general [15,17]; only two studies reported a decrease in the frequency of constipation [37] and bloating [46]. Six studies evaluated bowel habits without reporting significant changes [14,24,28,33,37,47].

*Stool characteristics.* Twenty studies analyzed stool pH, mass, frequency, consistency, and weight [9,11,14,16,21,22,24,25,28,29,32,33,37,38,39,40,41,43,45,46]. After DF intake, no significant changes were found in seven studies [9,16,24,25,29,32,43], while some studies reported an increase in: fecal hardness [39], fecal weight [9,11,40,45], stool frequency [22,41,45,46], fecal consistency [38], fecal softness and fecal water content [46], and formed stool [33]. On the other hand, a reduction in stool consistency and liquidity perceived [22], fecal dry matter [40], and fecal hardness [14] were also reported. Studies investigating changes in fecal pH after consumption of DFs [11,13,22,23,27,40] showed a reduction with the exception of two studies in which inulin and a WG-rich diet did not significantly change pH [14,43], respectively.

*Appetite.* This review did not find out any significant changes in appetite ratings [20,23,31,38] except for a decrease in satisfaction before lunch and hunger before dinner, and an increase in fullness and satisfaction after lunch when DFs were consumed 30 min before breakfast and 30 min before dinner [20].

*Gut hormones.* Some studies did not find changes in PYY [30,48], GLP-1 [31,48], and GLP-2 [31]. In contrast, the consumption of 15 g/day of AXOS for 12 weeks led to a decrease in postprandial GLP-1 AUC_0–90 min_ compared to placebo [38]; in addition, one study found an increase in GLP-1 after consumption of 40 g of high-amylose RS2 for 4 weeks [30]. Consuming 40 g of high-amylose RS2 for 4 weeks also led to increased secretion of C-peptide compared to control [30], while no changes were observed after consumption of 75 g of glucose combined with 24 g of inulin [19].

*Lipid profile*. Of the ten studies evaluating the lipid profile [17,26,30,35,37,40,43,45,47,48], no significant changes were found in seven [17,26,35,37,43,45,47], while three studies showed a reduction in certain blood lipid markers [30,40,48] with the exception of HDL that remained unchanged [40].

*Glucose and insulin*. Six out of seven studies did not find any significant changes [17,19,38,43,47,48], while increasing AXOS resulted in a dose-dependent decrease [31]. Regarding insulin response, one study reported significant treatment x time effects for post-prandial insulin concentrations [17] and another reported an increase at 30 min after meals [30]. In addition, DF consumption led to a reduction in ISI_composite_, i.e., the insulin sensitivity index, and no significant changes in HOMA-IR [31], while plasma insulin iAUC decreased with the consumption of 25.5 g/day of L-rhamnose compared to control [17]. In contrast, three studies did not find any significant changes [19,38,48].

*H_2_ breath excretion*. A significant increase was reported in four studies [17,19,30,35] after dietary intervention, while in two studies [18,38] DF intake did not influence H_2_ levels from baseline.

*Immune system.* Nine studies [14,15,22,32,33,37,38,45,48] also investigated the effects of DF intervention on immune-function-related markers: three studies reported no significant changes in immune parameters [14,32,48]; in one study [33], the response of two fecal immune markers (IgA and PGE_2_) was different, with the former significantly decreasing and the latter not varying. In other studies, DF intervention led to increased IgA expression [22] and a decrease in lymphocyte percentage in plasma [37]. One study [45] showed that DF intervention did not produce any significant effect on immune parameters except for a positive effect on effector memory T cells and acute innate immune response. In another study [15], the serum IL-4 level, the circulating percentages of CD282+/TLR2+ myeloid dendritic cells, and the ex vivo responsiveness to a toll-like receptor 2 agonist were higher, while the serum IL-10 level was lower after DF intervention.

*Gut permeability.* It was assessed in four studies [15,22,38,46]; two studies did not find any changes [38,46], while the other two found an increase [15] and a reduction [22] in circulating LPS after DF intervention.

*Other parameters*. After consuming 15 g/day of GOS for 4 weeks, one study reported no changes in oxidative stress parameters (malondialdehyde, trolox equivalent antioxidant capacity, and uric acid) [32]. Another study [23] investigated the influence of inulin (20 g/day for 4 weeks) on iron absorption in women with low-iron status, showing no change the iron status evaluated through erythrocyte incorporation of iron stable-isotope labels. On the other hand, inulin consumption was related to a significant increase in methane and a significant reduction in the rebound rate of free-fatty acids (FFA) compared to glucose consumption [19]. Another study evaluated FFA after consumption of 15 g/day of wheat bran extract for 12 weeks and reported no changes compared to placebo [38]. A WG diet was associated with higher excretion of nicotinurate in fecal waters and lower urinary excretion of carnitine, acetylcarnitine, urea, and taurine compared to a RG diet [44]. In addition, it was shown that the WG diet had a different effect between women (characterized by an increase in fumarate) and men (characterized by an increase in creatinine and a reduction in several metabolites, such as methylguanadine, pyruvate, citrate, succinate, 3-hydroxyisovalerate, and N-acetyl-glycoproteins). Another study evaluated delayed-type hypersensitivity (DTH response) after consuming a WG diet for 6 weeks and showed no significant changes [45]. Consumption of 15 g/day of arabinogalactan for 6 weeks increased the abundance of gut microbiome genes encoding α-l-rhamnosidase, β-fructosidase, and levanase, and the biosynthesis pathways for tricarboxylic acids and vitamin B6, compared to MD [24]. In contrast, one study showed that bacterial enzymatic activity decreased with the supplementation of XOS and INU-XOS compared to MD [22], while no significant changes in β-glucosidase were found after the consumption of 20 g/day of chicory inulin compared to control. Conversely, an increase in β-glucosidase activity was observed with NUTRIOSE^®^ at 10 and 15 g/day for 2 weeks compared to glucose [27]. Furthermore, one study found that consuming a low-FODMAP diet supplemented with OF at 14 g/day for 1 week led to a significant increase in colonic volume from baseline [35]. Regarding urinary metabolites, this DF intervention resulted in a significant decrease in aggregate metabolite score for carbohydrates and carbohydrate conjugates from baseline, while it did not change aggregate metabolite scores for amino acids, peptides and analogues, and lipids. Another study [38] evaluated the possible influence of supplementation with 15 g/day of wheat bran extract for 12 weeks on whole-gut transit time, oro-cecal transit time, and energy expenditure, and found that they were all unchanged compared to placebo. In addition, oro-fecal transit time decreased with LF [40]. Another study showed that consumption of 20 g/day of inulin for 3 weeks did not change intestinal transit time compared to control [14]. Systolic blood pressure was significantly lower after the supplementation of 25 g/day of LF and 25 g/day of CF for 4 weeks, compared to the control treatment [40]. After supplementation of 3×5 g/day of β2-1 fructan for 4 weeks [15], general wellbeing did not change while it decreased after supplementation of 6.64 g of INU-XOS for 3 weeks compared to MD [22]. No differences were found in the start and duration of fermentation with WB fractions compared to the control [18]. Reduced genotoxic effects of fecal waters were shown after PDX consumption [33], while no changes were found after interventions with WBE and OF [36]. On the other hand, consumption of WBE was associated with a significant decrease in fecal water cytotoxicity [36]. Finally, one study evaluated the effect of consumption of RS (40 g/day) for 4 weeks on liver function indices (i.e., aspartate aminotransferase, alanine aminotransferase, and γ-glutamyl transferase) and renal function (i.e., urea nitrogen), and found that all parameters remained unchanged except for a reduction in blood urea nitrogen [30].

## 4. Discussion

In this systematic review, we analyzed 42 randomized controlled trials reporting the effects of DFs on SCFA profile and, when available, on gut microbiota composition and other health outcomes. Studies were selected from a comprehensive database built to collect all the randomized, controlled, human trials performed on this topic and testing confirmed or candidate prebiotic components. We have found that DF interventions may affect the taxonomic community structure of the intestinal microbiota producing variable (non-univocal) significant effects on SCFA levels in apparently healthy adults. Among the 42 studies analyzed, 12 evaluated total SCFA; 7 of them showed that the consumption of DF led to a significant increase in total SCFAs, while no significant changes were observed in the other five studies. When considering individual SCFA levels, 26 studies did not report significant differences after the DF interventions, while significant changes for one or more SCFAs were described in the other 16 studies analyzed. Overall, these results underline that the available data are still not sufficient to definitely confirm the actual relationship between DF and SCFA production. Specifically, an important finding of this review is that the modulation of the SCFA profile is highly influenced by DF type, structure, and dose consumed, but also by individual characteristics (including gut microbiota composition).

It is generally recognized and supported by the literature that DFs can significantly modulate the microbiota composition that varied after the DF intervention compared to the control/placebo group; in our review, only three studies revealed no significant changes [9,43,46]. However, in 18 out of 42 studies analyzed the consumption of DFs led to modifications in the gut microbiota composition not accompanied by a significant modulation of the SCFA profile [10,12,16,20,21,23,24,25,27,28,32,33,35,38,39,47,48,49].

Thirteen studies included in this review [11,12,13,17,18,22,26,29,31,36,39,40,50] have demonstrated how diversified fiber structure can enrich diverse bacterial taxa. The structure of the carbohydrate provides competitive advantages both to bacterial strains and taxonomic groups in the colon: enzymes are produced by bacteria with a high specificity so that binding domains and catalytic site allow certain strains to cleave bonds, explaining the high strain-level diversity in the colon [71]. In this context, the concept of “discrete structures” has been developed and applied to DFs, defined as the unique chemical structures that align with gene clusters encoded in the genomes of specific microbial species, leading to a selective enrichment of a few bacteria [4]. This concept was supported by recent results also showing that small discrete differences in the structure of DF can differently influence the gut microbiota composition, as shown for the same category of fiber, type-IV resistant starch (RS4), for which discrete structural differences in chemical structure and granule size were sufficient to induce distinct effects on fecal microbiota composition and its functions [29]. Since the exact DF dose required for producing consistent outcomes in humans is unknown [72], another important variable we considered is the different amount tested for the same DFs in some studies analyzed. The results showed how higher DF doses can affect the intestinal microbial ecosystem more extensively than low doses [10,25,29,37,39]. These differences depend on the interaction between type and dose of DFs used [39].

The effects of DF interventions could be difficult to interpret due to the intricacy of the relationship between diet (which also includes a large number of variables per se) and gut microbiota composition by also considering the complexity of gut microbial metabolism. Metabolic cross-feeding is defined as the interaction between bacterial strains in which metabolites resulting from the metabolism of one strain are further metabolized by another strain [73]. Thus, metabolic cross-feeding could explain the observed taxonomic changes without a modification of the SCFA profile, and it is possible that the SCFAs could further shape the gut microbiota composition [74]. Another possible explanation is the utilization of SCFAs by the colonocytes for energy [7].

It has been shown that anthropometric variable (BMI), health/disease status, and age can have a great impact on the gut microbiota composition [75,76]. In order to limit the influence of these factors on the results after DF interventions, we included only studies performed in apparently healthy adults with an average BMI < 30 kg/m^2^ and a mean age between 18 and 60 years. However, there are further individual differences, which not all studies have considered, that can interfere with the interpretation of the results on the impact of DF intervention on gut microbiota and derived metabolites. In particular, sex could be a factor influencing the gut microbiota [75] and SCFA-related results, but only two studies have considered sex-specific effects in the evaluation of results [26,44]. Specifically, one of the studies [26] reported that the final fecal metabolome was mostly affected by inter-individual (e.g., in blood and anthropometric parameters) and sex differences (∼28% variation and ∼15% variation, respectively), while the DF treatment affected only ∼8% of variation. In particular, a more consistent effect on SCFA profile was observed in males compared to females [26].

It is noteworthy to consider that the interest in DFs-dependent SCFA production, also explored by the present review, is linked to the potential direct or indirect role played by these microbial metabolites on function and biological activities linked to human health. For example, a possible mechanism through which SCFAs can provide health benefits is related to the reduction in colonic and fecal pH. A decrease in gut pH could be associated with a reduction in *Clostridium perfrigens*, a potentially harmful bacterium, thus promoting a more favorable and healthy gut environment [27].

Among the beneficial effects that SCFAs could have on the host, there are many proposed favorable metabolic impacts which seem to support the important contribution of fiber intake for the reduction in numerous chronic diseases. For example, the production of SCFAs during colonic fermentation of indigestible carbohydrates has been reported to be associated with beneficial effects on glucose metabolism. The underlying mechanism is not clear, but it has been suggested that there is a strong correlation between circulating FFAs, insulin sensitivity [76], and glucose tolerance [77,78,79]. These effects could also support in part the hypothesized role of DF-derived SCFAs on body weight and eating behavior mediated by gut hormone levels and appetite/satiety sensation [30]. For example, propionate has been related to an amelioration of satiety [80] through an induction in anorectic hormones and intestinal gluconeogenesis, which also affects glucose metabolism [81,82,83].

Moreover, SCFA production is supposed to promote a functional gut intestinal barrier, which is pivotal for reducing intestinal permeability found in many different diseases [84,85]. In this regard, animal models have revealed a beneficial role of butyrate in maintaining gastrointestinal barrier integrity, quenching oxygen at the epithelial interface, and exerting immune-modulating [86,87] and also anti-carcinogenic effects, even if direct evidence in humans is still limited [88].

While the health promoting effect of dietary fiber is well recognized and acknowledged [89], the mixed results obtained in the present review specifically focused on the impact of DF interventions on SCFA production and gut microbiota composition cannot help providing consistent and definitive confirmation of the extent of such an impact, at least in healthy adults. This may be due to the paucity of available results and the numerous factors affecting response to a treatment by also considering that, in most of the cases, the analysis of health-related biomarkers was performed as secondary or even tertiary outcomes; thus, results may be critical or unreliable due to an inadequate sample size used to test the hypothesis of a significant modulatory effect.

It is also noteworthy that fecal concentration of SCFAs may not directly represent the rate of their production due to several factors such as the rapid absorption of most SCFAs produced in the large intestine [90]. The last but not the least, the analytical method adopted to evaluate SCFA levels in biological samples was found to be critical in providing accurate data, consequently affecting the results obtained, as discussed critically in a recent report [91], and increasing uncertainties in the assessment of such studies.

Finally, the lack of adequate control for total DF intake observed in many studies (see Supplementary Table S3) deserves a major comment, in that often only the amount of single fibers added to the diet has been considered and the two treatments are not always comparable for total DF intake. This limitation could conceal a total amount of daily fiber even largely below those generally recommended.

## 5. Conclusions and Future Perspectives

Despite the large interest on prebiotic dietary fibers and their positive impact on human health, the results of the present review indicate that, due to the lack of univocal data obtained, it is still difficult to define how, how much, to whom, and which dietary fibers should be suggested to affect the intestinal microbial ecosystem in healthy adults. In addition, the results are marred by significant heterogeneity in analytic methods used to analyze SCFA profile across the available studies, suggesting the need for standardized/harmonized protocols to obtain results that can be compared.

High-quality RCTs are needed to clarify the effects of DF intervention on SCFA profile and gut microbiota composition in order to better understand the possible role of specific categories of dietary fiber, as key compounds exerting possible targeted health benefits. In particular, the future studies should be performed by considering different important aspects. First, a careful selection, standardization, and characterization of the study population should be planned in order to limit the presence of potential confounding factors that are able to increase inter-individual variability. Second, standardized procedures and analytical methodologies should be adopted to provide accurate results that facilitate the comparison between studies. Third, the impact/role/contribution of the different DFs, DF-food sources, food matrices, and degree of DF polymerization need to be evaluated. Fourth, dose–response studies that also take into consideration the contribution of time exposure should be carried out. Fifth, when analyzing data, the contribution of the overall diet (and fiber intake specifically) in the modulation of gut microbiota, SCFAs, and health-related markers should be carefully considered. In addition, other important factors to consider are adequate power calculation, randomization, and identification of the best control/placebo treatment and evaluation of baseline individual characteristics, including microbiota composition.

In the near future, the possibility to draw conclusions on concordant data based on the same methodological workflow will allow researchers to delve into specific elaborations (e.g., helping to define prebiotic role of dietary compounds in health and disease, defining specific prebiotic recommendations) based on unbiased and enlarged reference datasets with a clear and appropriate statistical power.

## Figures and Tables

**Table 1:** PICOS table for inclusion of studies.

Parameter	Inclusion Criteria
Population	Age 18 to 60 years and BMI < 30 kg/m^2^
Intervention	Dietary intervention studies involving the consumption of dietary fiber, supplied with food or supplements, without probiotics and/or synbiotics
Comparison	Control group
Outcome	At least SCFA profile
Study design	Human clinical and randomized controlled trials

## Data Availability

Not applicable.

## Supplementary Materials

The following are available online at https://www.mdpi.com/article/10.3390/nu14132559/s1, Table S1: Characteristics of the dietary intervention studies investigating the effects of dietary fibers consumption. Table S2: Daily fiber intake of participants during the DF interventions reported by studies included in the review. Table S3: Characteristics of the health-related outcomes and their findings. Figure S1: Risk of bias for each item assessed in each of the included studies. Figure S2: Risk of bias for each item assessed, presented as a percentage across all included studies combined.
