# Supplementary material for: Effects of Dietary Fibers on Short-Chain Fatty Acids and Gut Microbiota Composition in Healthy Adults: A Systematic Review
Source: Nutrients. 2022 Jun 21;14(13):2559. doi: 10.3390/nu14132559 (PMC9268559; doi:10.3390/nu14132559)
Supplement: Supplementary file 1 [file nutrients-14-02559-s001.zip › Suppl_Figures S1and S2.pdf]

	(1) random sequence generation	(2) allocation concealment	(3) blinding of participants and personnel	(4) blinding of outcome assessment	(5) incomplete outcome data	(6) selective reporting data	(7) other bias
Brandl et al. 2020	+	+	+	?	-	+	?
Reimer et al. 2020	+	+	+	+	-	?	?
Boler et al. 2011	+	?	+	+	+	+	?
Fernando et al. 2010	?	?	-	-	+	?	?
Hooda et al. 2012	?	?	+	+	+	+	?
Slavin et al. 2011	?	?	+	?	+	+	?
Clarke et al. 2016	+	+	+	+	?	-	?
Costabile et al. 2010	?	?	+	+	?	+	?
Darzi et al. 2016	+	+	?	-	?	-	?
Deroover et al. 2017	+	+	+	?	+	+	?
Fernandes et al. 2011	?	?	?	?	+	+	?
Healey et al. 2018	+	+	+	+	+	-	?
Holscher et al. 2015	?	?	+	+	+	-	?
Lecerf et al. 2012	?	?	+	?	?	-	?
Petry et al. 2012	?	?	+	?	+	+	?
Chen et al. 2021	+	+	+	+	?	+	?
Finegold et al. 2014	?	?	+	?	-	+	?
Trimigno et al. 2017	?	?	?	+	?	-	?
Lefranc-Millot et al. 2012	-	-	+	?	-	-	?
Alfa et al. 2018	+	+	+	+	-	+	?
Deehan et al. 2020	+	+	+	+	?	+	?
Zhang et al. 2019	+	+	+	+	?	?	?
Boll et al. 2016	?	?	-	-	-	?	?
Wilms et al. 2021	+	+	+	+	-	-	?
Costabile et al. 2012	-	-	+	+	?	+	?
Lamichhane et al. 2014	-	-	+	+	+	+	?
Sloan et al. 2018	?	?	+	+	?	+	?
Windey et al. 2014	+	+	+	?	?	+	?
François et al. 2012	+	+	+	+	?	?	?
Müller et al. 2020	+	+	+	?	-	+	?
Elison et al. 2016	+	+	+	?	?	-	?
Fechner et al. 2014	+	+	+	+	+	+	?
Damen et al. 2012	?	?	+	?	+	+	?
Walton et al. 2012	?	?	+	?	-	?	?
Ampatzoglou et al. 2015	-	-	-	?	+	+	?
Ross et al. 2013	?	?	-	?	+	+	?
Vanegas et al. 2017	+	+	-	?	-	-	?
Vuholm et al. 2017	+	+	-	?	-	-	?
Carvalho-Wells et al. 2010	?	?	+	?	-	?	?
Connolly et al. 2016	+	+	+	?	-	+	?
Duysburgh et al. 2021	?	?	-	?	?	?	?
Nilsson et al. 2010	?	?	-	?	+	+	?

Supplementary Figure S1: Risk of bias for each item assessed in each of the included studies

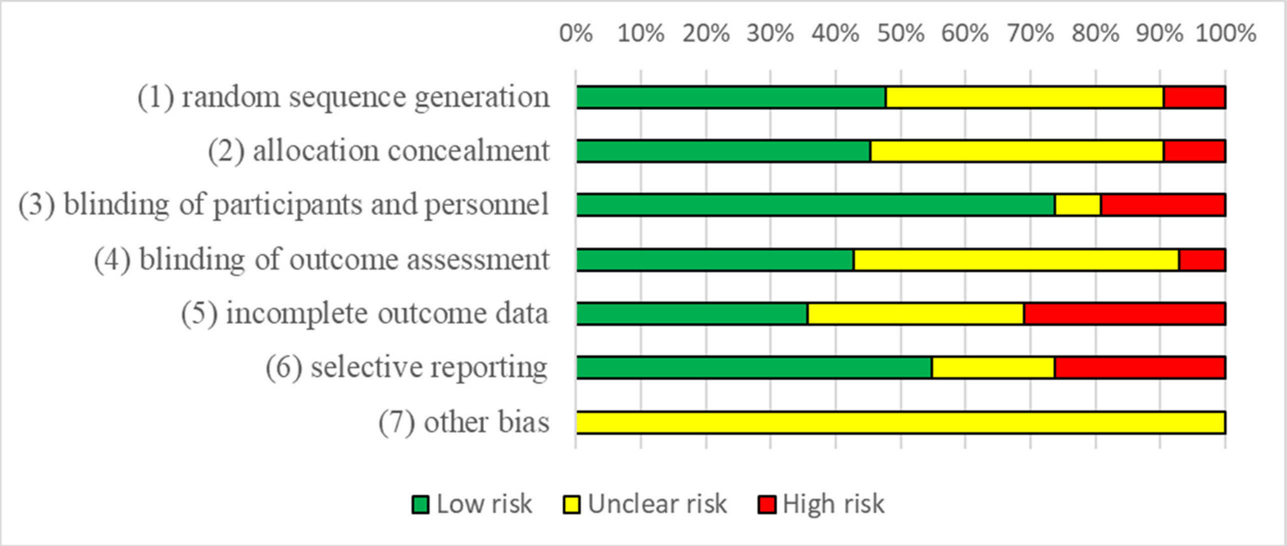

Supplementary Figure S2: Risk of bias for each item assessed, presented as a percentage across all included studies combined
