# Supplementary material for: Effects of Dietary Fibers on Short-Chain Fatty Acids and Gut Microbiota Composition in Healthy Adults: A Systematic Review
Source: Nutrients. 2022 Jun 21;14(13):2559. doi: 10.3390/nu14132559 (PMC9268559; doi:10.3390/nu14132559)
Supplement: Supplementary file 1 [file nutrients-14-02559-s001.zip › Suppl_Table S1.pdf]

Table S1. Characteristics of the dietary intervention studies investigating the effects of dietary fibers consumption.

Reference	Study Population[a]	Study Design	Prebiotic intervention[b]	Control/ Placebo Intervention[b]	Short-Chain Fatty Acids Analysis[c]	Fecal Microbiota Analysis[c]	Microbiota-Related Findings
[28]	42 mid-age adults (18 M, 24 F)<br>Age: 41.6 ± 5.6 years<br>BMI: N.A.	Randomized, prospective, placebo-controlled, parallel, double-blind trial	30 g/day of RS (70% RS2) for 3 months	30 g/day of placebo (fiber content n.d.) for 3 months	SCFAs by GC in fecal samples	16S rRNA amplicon sequencing (V4 hyper-variable region)	No significant changes in SCFAs with RS vs. baseline, Amioca TF<br><br>↓ alpha diversity (Shannon and Inv. Simpson) and Firmicutes/Bacteroidetes ratio with MSPrebiotic® RS<br>↑ *Bifidobacterium* with RS vs. Amioca TF<br>↑ *Bifidobacterium* (↑ *B. ruminantium* and ↓ *B. dentium*) and *Olsenella* with RS<br>↓ *Coprobacillus* and *Lactobacillus* with RS vs. Amioca TF<br>↑ *Ruminoccocus bromii*, ↓ *R. obeum* and *R. torques* with RS
[43]	33 healthy adults (12 M, 21 W)<br>Age: 48.8 ± 1.1 years<br>BMI: 27.9 ± 0.7 kg/m^2	Randomized, controlled, cross-over trial<br><br>Washout (4 weeks)	Diets high in WG (>80 g/day) (fiber content n.d.) for 6 weeks	RG diet low in WG (<16 g/day) (fiber content n.d.) for 6 weeks	SCFAs, BCFAs by GC in fecal samples	FISH: total bacteria, *Clostridium coccoides*/*Eubacterium rectale* group, *Bifidobacterium*, *Lactobacillus*/*Enterococcus* group, *Bacteroides*/*Prevotella* group, *Clostridium histolyticum* group and *Escherichia coli*	No changes in total and individual SCFAs, BCFAs with WG vs. baseline, RG<br><br>No changes in the gut microbiota
[11]	21 healthy adult men<br>Age: 27.5 ± 4.3 years<br>BMI: 27.0 ± 4.0 kg/m^2	Randomized, double-blind, placebo-controlled, cross-over trial<br><br>No washout	1) 21 g/day of PDX in 3 snack bars/day for 21 days<br><br>2) 21 g/day of SCF in 3 snack bars/day for 21 days	3 snack bars/day without supplemental fiber (NFC) for 3 weeks	SCFAs and BCFAs by GC in fecal samples	qPCR: *Escherichia coli*, *Bifidobacterium* and *Lactobacillus*	↓ Acetate, propionate and butyrate with PDX vs. SCF, NFC<br>↓ Valerate with PDX vs. NFC<br>↑ Acetate molar ratio, ↓ butyrate molar ratio with PDX, SCF vs. NFC<br>↓ Propionate molar ratio (*p*=0.09) with PDX vs. NFC<br>↓ BCFAs with PDX, SCF vs. NFC<br><br>↑ *Bifidobacterium* spp. with SCF vs. NFC

[31]	19 healthy adults (9 M, 10 F)<br>Age: 23,0 ± 0.4 years<br>BMI: 22.2 ± 0.4 kg/m^2	Randomized, controlled, cross-over trial<br><br>Washout (approximately 1 week)	1) WWB + AXOS + RS: 8.9 g AXOS ww + 1.5 g of Glucans ww + 6.6 g RS ww (from a 140.5 g portion sizes of the test products) for one late evening meal<br><br>2) WWB + increased content of AXOS (hiAXOS): 18.4 g AXOS ww + 3.1 g glucans ww + 1.0 g RS ww (from a 141.4 g portion sizes of the test products) for one late evening meal<br><br>3) WWB + increased content of RS (hiRS): <0.15 g Glucans ww + 15 g RS ww (from a 137.9 g portion sizes of the test products) for one late evening meal	1) Glucans: <0.15 g/portion (wet weight) + RS: 1.2 g/portion (wet weight)<br>for one late evening meal	SCFAs measured by GC in plasma samples	N.A.	↑ Total SCFAs, acetate with AXOS vs. WWB<br>↑ Butyrate with WWB + hiAXOS vs. WWB<br>Significant time × treatment interaction for butyrate<br>↑ Linear dose-dependent in circulating acetate, butyrate and total SCFAs at fasting and during the postprandial period with increasing content of AXOS<br>↑ Circulating total SCFAs at fasting significantly related to ↓ GLU response in the later postprandial phase<br>↑ Breath H_2 and total SCFAs during the experimental day strongly related
[9]	20 adults (8 M, 12 F)<br><br>TRIAL 1<br>Age: 25.0 ± 4.0 years<br>BMI (control): 22.4 ± 1.9 kg/m^2<br>BMI (prebiotic): 22.6 ± 2.2 kg/m^2<br><br>TRIAL 2<br>Age: 24.0 ± 4.0 years	TRIAL 1<br>Randomized, placebo-controlled, double-blind, cross-over trial<br><br>TRIAL 2<br>Randomized, placebo-controlled, double-blind, cross-over trial<br><br>Washout (3 days)	TRIAL 1<br>Food products enriched with 10 g extrinsic wheat fiber<br>for 5 days<br><br>TRIAL 2<br>Drinks enriched with 10 g extrinsic wheat fiber (wheat plant fibre 3.75%, psyllium 3.75%, guar 3.75%, from powder dissolved in 300 mL water)<br>for 5 days	TRIAL 1<br>Same products without added fiber (fiber content n.d.)<br>for 5 days<br><br>TRIAL 2<br>Placebo powdered cellulose 3.75%; psyllium 3.75%; guar 3.75%<br>for 5 days	VFAs, 2-methyl butyrate and 4-methyl valerate by LC-MS/MS in fecal samples	16S rRNA amplicon sequencing (target region not stated)	No changes in SCFAs with PRE (both trials) vs. CTRL<br>↑ Acetate with TRIAL 1 vs. TRIAL 2<br><br>No alterations in gut microbiota profiles

	BMI (control): 22 ± 2.0 kg/m^2<br>BMI (prebiotic): 22 ± 2.0 kg/m^2						
[47]	32 healthy adults (11 M, 21 F)<br>Age: 31.6 ± 8.0 years<br>BMI 23.3 ± 0.6 kg/m^2	Randomized, placebo-controlled, double-blind, cross-over trial<br><br>Washout (3 weeks)	48 g/day of WGM (total fiber 14.2 g) for 3 weeks	48 g/day of NWG (total fiber 0.81 g) for 3 weeks	SCFAs, BCFAs, lactic and valeric acids by HPLC in fecal samples	FISH: total bacteria, *Bifidobacterium* spp., lactobacilli/enterococci, *Clostridium perfringens/histolyticum* subgroup, *Bacteroides* groups, members of *Clostridium* cluster XIVa, *Roseburia/Eubacterium rectale* group and *Atopobium* spp.	No changes in SCFAs according to treatment or in washout<br><br>↑ *Bifidobacterium* spp.<br>After a 3-week washout period, bifidobacterial levels returned to pre-intervention levels
[24]	30 healthy adults (14 M, 16 F)<br>Age: 41.1 ± 9.5 years<br>BMI: 26.4 ± 3.4 kg/m^2	Randomized, placebo-controlled, double-blind, cross-over trial<br><br>Washout (3 weeks)	15 g/day of arabinogalactan for 6 weeks	15 g/day of MD (fiber content n.d.) for 6 weeks	SCFAs, BCFAs and hexanoic acid by GC-MS in fecal samples	16S rRNA amplicon sequencing (V3-V4 hyper-variable regions)	↓ Isovaleric, valeric and hexanoic acid with arabinogalactan vs. MD<br>No differences for SCFAs and isobutyrate with arabinogalactan vs. MD<br><br>- α-diversity:<br>↓ Shannon, ↓ Simpson, ↓ Chao1 (p=0.082)<br><br>- Taxa:<br>↓Firmicutes/Bacteroidetes ratio, ↑ Bacteroidetes, ↓ Firmicutes, ↑ *Bifidobacterium* (p=0.088), ↑ [*Eubacterium*] *ruminantium* group, ↑ *Eisenbergiella*, ↑ *Howardella*, ↑ GCA-900066225 genus (uncultured *Ruminococcus*), ↑ DTU089 genus (methane producer), ↓ CAG-352 genus (*Ruminococcaceae*), ↓ *Victivallis*

[15]	30 healthy adults (13 M, 17 F) Age: 28.1 ± 5.1 years BMI: 24.2 ± 3.0 kg/m^2	Randomized, placebo-controlled, double-blind, cross-over trial<br>Washout (2 weeks)	3×5 g/day of β2-1 fructan (50:50 mixture of IN and short-chain oligosaccharides) for 4 weeks	3x5 g/day of MD (fiber content n.d.) for 4 weeks	SCFAs and BCFAs by GLC in fecal samples	qPCR: eubacteria and *Bifidobacterium* spp.	↑ Total SCFAs with Orafti vs. MD (↑proportion of propionate and butyrate ↓ acetate ↓ proportions of BCFAs except for heptanoic acid)<br>↑ *Bifidobacterium* spp.
[48]	30 adults mildly hypercholesterolemic or glucose intolerant (11 M, 19 F) Age: 42 (SD N.A.) years BMI: 26.4 ± 5.7 kg/m^2	Randomized, double-blind, controlled, cross-over trial<br>Washout (4 weeks)	45 g/day of WGO breakfast cereal (~ 2.8 g fiber and 1.3 g -glucan) for 6 weeks	45 g/day of NWG (~1.35 g fiber) breakfast cereal for 6 weeks	SCFAs by HPLC in fecal samples	FISH: *Bifidobacterium* spp., *Bacteroides/Prevotella* spp., *Lactobacillus/ Enterococcus* spp., *Clostridium coccoides/Eubacterium rectale* group, *Clostridium histolyticum* group, and *Atopobium* cluster including most *Coriobacteriaceae* spp.	No significant changes in SCFAs with WGO vs. baseline, NWG<br>↑*Bifidobacterium,* lactobacilli, and total bacteria with WGO
[16]	32 healthy adults (14 M, 18 F) Age: 25 years (SD N.A.) BMI: N.A. (inclusion criterion 20-30 kg/m^2)	Randomized, placebo-controlled, double-blind, cross-over trial<br>Washout (3 weeks)	10 g/day of VLCI derived from globe artichoke (inulin: 97.5 %) for 3 weeks	10 g/day of MD (fiber content n.d.) for 3 weeks	SCFAs by HPLC in fecal samples	FISH: total bacteria, *Bifidobacterium* spp., *Lactobacillus* spp., *Bacteroides*, *Prevotella*, *Escherichia coli*, *Clostridium* and *Ruminococcus*	No significant changes in SCFAs with VLCI vs. baseline, MD<br>↑ *Bifidobacterium* spp.<br>↑ *Lactobacillus–Enterococcus*<br>↑ *Atopobium*<br>↓ *Bacteroides–Prevotella*
[33]	31 healthy adults (15 M, 16 F)	Randomized, placebo-controlled,	8 g/day of PDX powder for 3 weeks	8 g/day of MD powder (fiber content n.d.)	SCFAs, BCFAs and n-caproid	FISH and qPCR: total bacteria, *Atopobium* group,	No changes in SCFAs, BCFAs and n-caproid acid with PDX vs. baseline, MD

	Age: 33 years (SD N.A.)<br>BMI: 24.1 ± 2.8 kg/m²	double-blind, cross-over trial<br><br>Washout (3 weeks)		for 3 weeks	acid by GC in fecal samples	*Bacteroides* spp., *Bifidobacterium* spp., *Eubacterium rectale/Clostridium coccoides* group, *Clostridium histolyticum* group, *Lactobacillus/Enterococcus* spp., and *Faecalibacterium prausnitzii*	FISH and qPCR<br>↑ *Ruminococcus intestinalis, Clostridium* clusters I, II and IV (*C. leptum* group), *E. rectale-C. coccoides* group with PDX (the latter also with MD)<br><br>↓ *C. histolyticum* (clusters I and II) and *Lactobacillus-Enterococcus* group with PDX (the latter also with MD)
[41]	27 healthy adults (10 M, 17 F)<br>Age: 25 years (median)<br>BMI: 20.9 kg/m² (median)	Randomized, double-blind, controlled, cross-over trial<br><br>Washout (3 weeks)	180 g of wheat/rye bread with *in situ* produced AXOS (4.0% AX dm + 2.14 g AXOS + 9.2 % total fiber) (WR+)<br>for 3 weeks	180 g of RW flour bread without AXOS (3.7% AX dm + 10.1 % total fiber) (WR-)<br>for 3 weeks	SCFAs by GC in fecal samples	FISH: total bacteria, *Bifidobacterium* spp., *Lactobacillus* spp., enterobacteria, *Clostridium histolyticum/litusebu rense* group	↑ Total SCFAs, butyrate with WR+ vs. pre-WR+<br>↑ Acetate, propionate with WR+ vs. pre-WR+ (not significant)<br><br>↑ *Bifidobacterium* spp. (*p*=0.06)
[17]	13 healthy adults (5 M, 8 F)<br>Age: 23.0 ± 4.0 years<br>BMI: 22.1 ± 1.6 kg/m²	Single-blind, randomized, controlled, cross-over trial<br><br>Washout (≥1 week)	1) 25.5 g/day L-Rha<br>2) 22.4 g/day IN<br>alongside a standardized breakfast and lunch	Standardized breakfast and lunch without supplement (fiber content n.d.)	SCFAs by GC in serum samples	N.A.	No significant changes in SCFAs between treatments
[29]	40 healthy adults (20 M, 20 F)<br>Age: 28.4 ± 8.1 years<br>BMI: 24.0 ± 3.2 kg/m²	Randomized, placebo-controlled, parallel four-arm dose-response, double-blind, trial	1) 10, 20, 35 and 50 g/day of maize RS4 (65.0/58.2 dwb/as-is % total fiber)<br>2) 10, 20, 35 and 50 g/day of potato RS4 (90.0/78.7 dwb/as-is % total fiber)<br>3) 10, 20, 35 and 50 g/day of tapioca RS4 (96.0/85.3 dwb/as-is % total fiber)<br><br>for 4 weeks	10, 20, 35, and 50 g/day of digestible corn starch (0.0 g of fiber)<br>for 4 weeks	SCFAs and BCFAs by GC-MS in fecal samples	16S rRNA amplicon sequencing (V5-V6 hyper-variable regions)	No significant changes in SCFAs with RS4 vs. baseline<br>↑ Butyrate concentration and relative proportion, ↓ Propionate relative proportion with maize RS4 vs. baseline<br>↑ Propionate with tapioca RS4 at dose 35 g/day vs. baseline<br>↑ Propionate with tapioca RS4 vs. maize RS4<br>↓ BCFAs to SCFAs ratio with maize RS4 vs. baseline<br>↓ BCFAs to SCFAs ratio with tapioca RS4 vs. potato RS4, PLA

			to achieve a gradual increase to 10, 20, 35, and 50 g/day.				- α-diversity: dose-dependent ↓ Shannon and ↓ Pielou Evenness for maize and tapioca RS4s<br>= richness (total OTUs)<br>- β-diversity: maize and tapioca RS4s increased inter-subject variation (Bray-Curtis dissimilarity). NMDS scaling analysis showed differences between individuals consuming maize and tapioca RS4s when compared with baseline and PLA.<br><br>- Taxa:<br>• for maize RS4 - ↑ *Eubacterium rectale*, *Oscillibacter* spp., *Ruminococcaceae*<br>↓ *Ruminococcus callidus*, ↓ *Agathobaculum butyriciproducens* ↓ *Adlercreutzia equolifaciens*<br>• for tapioca RS4 - ↑ *Porphyromonadaceae*, ↑ *Parabacteroides*, ↑ *Parabacteroides distasonis*, ↑ *Parabacteroides* spp., ↑ *Faecalibacterium prausnitzii*, ↑ *Eisenbergiella* spp. ↓ unclassified *Ruminococcaceae*, ↓ *Eubacterium hallii*, ↓ *Clostridium viride*<br>• No effects detected for potato RS4 and PLA.<br><br>- Other: the observed effects were dose-dependent with a plateau at 35 g.
[18]	10 healthy adults (4 M, 6 F)<br>Age: 25.0 ± 4.0 years<br>BMI: 23.7 ± 2.0 kg/m^2	Randomized, controlled, cross-over trial<br><br>Washout (at least 1 week)	Standard breakfast with:<br>1) 10 g ^{13}C-inulin + 10 g inulin-^{14}C-carboxylic acid + 20 g unmodified WB fraction (50% fiber)<br>2) 10 g ^{13}C-inulin + 10 g inulin-^{14}C-carboxylic acid + 20 g WB-RPS (50% fiber)<br>3) 10 g ^{13}C-inulin + 10 g inulin-^{14}C-carboxylic acid + 20 g PE-WB (71% fiber)	Standard breakfast without wheat bran fraction (10 g 13C-inulin + 10 g inulin-14C-carboxylic acid)	^{13}C-labeled SCFAs by GC in plasma samples	N.A.	No changes in cumulative ^{13}C-SCFA concentrations and in the relative proportions of acetate, propionate, and butyrate with WB fractions vs. CTRL
[49]	34 healthy adults with mild hypercholes terolemia	Randomized, single-blind, placebo-controlled, cross-over	40 g/day oats containing 1.4 g β-glucans for 6 weeks	40 g/day cream of rice (o g of fiber)<br>for 6 weeks	SCFAs by UPLC-MS/MS in plasma samples	qPCR: *Lactobacillus* spp. and *Bifidobacterium* spp.	No significant changes in SCFAs with β-glucans vs. CTRL<br><br>↑ *Lactobacillus* spp.<br>↑ *Bifidobacterium* spp. (not significant)

	Age: N.A. (range 18-65 years)<br>BMI: N.A. (inclusion criterion 18.5-30 kg/m²)	trial<br><br>Washout (4 weeks)					
[39]	100 healthy adults<br><br>Glucose group<br>Age: 34.9 ± 6.3 years<br>BMI: 26.8 ± 6.3 kg/m²<br><br>Intervention group<br>Age: 36.1 years<br>BMI: 25.2 kg/m²	Randomized, placebo-controlled, double-blind, parallel trial	1) 5, 10 or 20 g/day of 2′FL<br>2) 5, 10 or 20 g/day of LNnT<br>3) 5, 10 or 20 g/day of 2′FL+LNnT (2:1 mass ratio; mix)<br>for 2 weeks	2 g/day of glucose (fiber content n.d.)<br>for 2 weeks	SCFAs by HPLC in fecal samples	16S rRNA amplicon sequencing (V3-V4 hyper-variable regions)	No significant changes in SCFAs after 2 weeks of intervention<br><br>↑ Actinobacteria with LNnT at all doses vs. baseline, ↓ Firmicutes with 20 g, ↓ Proteobacteria with 10g<br><br>↑ Actinobacteria with 2′FL at 5 g and 10 g vs. baseline<br><br>↑ Actinobacteria with mix at 10 g and 20 g vs. baseline, ↓ Firmicutes with 20 g<br><br>↑ *Bifidobacterium*, and 3 OTUs (*B. adolescentis*, *B. longum*, and *B. bifidum*) after intervention
[19]	9 adults (4 M, 5 F) with normal insulin levels<br>Age: 35.9 ± 3.8 years<br>BMI: 25.4 ± 1.5 kg/m²	Single-blind, randomized, controlled, cross-over trial<br><br>Washout (13 ± 2 d)	75 g GLU + 24 g Inulin (IN) on one morning	75 g GLU (fiber content n.d.) on one morning	SCFAs by GC in serum samples	N.A.	No changes in SCFAs with IN vs. GLU<br>↑ Serum acetate (4 h) in normal-insulin subjects vs. high-insulin subjects<br>A significant time × treatment interaction for serum acetate
[12]	12 healthy adults (7 M, 5 F)<br>Age: 25.6 ± 8.7 years<br>BMI: N.A.	Randomized, controlled, cross-over trial<br><br>No washout	Usual diet supplemented with:<br>1) 200 g/day of canned chickpeas (expected to contain 3-5 g of oligosaccharides) for 3 weeks<br>2) 5 g/day of raffinose for 3 weeks	Usual diet without supplementation (fiber content n.d.)<br>for 3 weeks	SCFAs, BCFAs and caproic, eptanoic iso-caproic and lactic acids by	T-RFLP analysis (various microbial groups)<br>qPCR: *Clostridium coccoides - Eubacterium rectale*	No changes in total SCFAs or individual SCFAs among the three diets<br><br>T-RFLP and qPCR<br>↓ number of individuals showing *Clostridium histolyticum*/*Clostridum lituseburense* groups and ↓

					GC in fecal samples	group, *Clostridium leptum* subgroup, *Bifidobacterium*, Bacteroidetes, lactobacilli	ammonia-producing bacteria with the chickpea diet. Short-term dietary intervention may have a limited capacity for modifying colonic microbiota in the longer term.
[40]	52 moderately hypercholes terolemic adults (20 M, 32 F) Age: 46.9 ± 3.2 years BMI: 26.5 ± 5.9 kg/m²	Randomized, double-blind, controlled, cross-over trial<br><br>Washout (2 weeks)	High-fiber diet with:<br>1) 25 g/day of LF for 4 weeks<br>2) 25 g/day of CF for 4 weeks	Low-fiber control diet without added fiber (CD) (fiber content n.d.) for 4 weeks	SCFAs by GC-FID in fecal samples	N.A.	↑ Total SCFAs, SCFAs with LF vs. baseline, CD<br>↑ Acetate with CF vs. CD<br>↑ Total SCFAs, acetate with CF vs. baseline<br>Negative correlation between changes in LDL and changes in SCFA excretion
[25]	30 healthy adults (11 M, 21 F)<br><br>Age: 30.1 years (SD N.A.) (M) BMI: 25.6 kg/m² (SD N.A.) (M)<br><br>Age: 33.6 years (SD N.A.) (F) BMI: 24.1 kg/m² (SD N.A.) (F)	Randomized, placebo-controlled, parallel, double-blind, trial<br><br>Washout (2 weeks)	1) 175 mg/day of XOS (from a capsule of 1.4 g) XOS for 8 weeks<br>2) 350 mg/day of XOS (from a capsule of 2.8 g) XOS for 8 weeks	Capsule placebo for 8 weeks	SCFAs (volatile and non-volatile) by GLC in fecal samples	Culture-based analysis: total aerobic and anaerobic counts, *Enterobacteriaceae*, *Bacteroides fragilis* group, *Clostridium*, *Bifidobacterium*, and *Lactobacillus*<br>454 Pyrosequencing	No significant changes in SCFAs with XOS vs. baseline, PLA<br><br>Culture-based analysis<br>↑ *Bifidobacterium*, ↑ total anaerobes, ↑ *Bacteroides fragilis* group with XOS<br><br>454 Pyrosequencing<br>↑ *Faecalibacterium*, ↑ *Akkermansia* after high-dose XOS
[37]	57 healthy adults (30 M, 27 F) Age: 40.7 ± 2.2* years	Randomized, placebo-controlled, cross-over trial	1) Treatment with WBE at 3 g/day (2.4 g AXOS/day) for 3 weeks<br>2) Treatment with WBE/day at 10 g/day (8 g AXOS/day) for 3 weeks	Placebo drink (70 ml twice daily) (fiber content n.d.) for 3 weeks	SCFAs by GC in fecal samples	FISH: total bacteria, *Bifidobacterium*, *Lactobacillus/Enterococcus*, *Clostridium histolyticum/*	↑ Total SCFAs, acetate, propionate, butyrate with WBE at 10 g/day vs. PLA<br>↑ Propionate with WBE at 3 g/day vs. PLA<br>↑ Total SCFAs, acetate and propionate as overall WBE effects

	BMI: 23.5 ± 0.4* kg/m²	Washout (2 weeks)				*lituseburense, Faecalibacterium prausnitzii, Roseburia/Eubacterium rectale*	↑ *Bifidobacterium* with WBE at 10 g/day vs. PLA
[20]	34 healthy adults<br><br>Low dietary fiber, LDF (6 M, 8 F) Age: 37.7 ± 10.6 years BMI: 24.3 ± 2.7 kg/m²<br><br>High dietary fiber, HDF (7 M, 13 F) Age 37.2 ± 14.4 years BMI: 22.5 ± 2.8 kg/m²	Randomized, double-blind, placebo-controlled, cross-over trial<br><br>Washout (3 weeks)	16 g/day of powdered inulin-type fructan prebiotic (50:50 inulin to fructo-oligosaccharide mix;) as two 8 g/day doses for 3 weeks	16 g/day of MD (fiber content n.d.) for 3 weeks	SCFAs by GC in fecal samples	16S rRNA amplicon sequencing (V3–V4 hyper-variable regions)	No significant changes in SCFAs with Orafti vs. baseline<br><br>- α-diversity:<br>↓ Shannon (p=0.06) in the HDF group<br>↑ Chao1 in the LDF group<br><br>- Taxa: in all volunteers:<br>↑ Actinobacteria, ↓ Firmicutes, ↓ Proteobacteria (p=0.07); ↑ *Bifidobacterium*, ↓ *Coprococcus*, ↓ *Dorea*, ↓ *Ruminococcus*, ↓ *Oscillospira*, ↑ *Faecalibacterium* (p=0.088)<br><br>In the HDF group: ↑ Actinobacteria, ↓ Firmicutes, ↑ *Bifidobacterium*, ↑ *Faecalibacterium*, ↓ *Coprococcus*, ↓ *Dorea*, ↓ *Ruminococcus*<br>In the LDF group: ↑ Actinobacteria, ↑ *Bifidobacterium*
[21]	29 healthy adults (14 M, 15 F) Age: 27 ± 4.1 years BMI: 24.4 ± 2.3 kg/m²	Randomized, placebo-controlled, double-blind, cross-over trial<br><br>Washout (1 week)	1) 5.0 g of agave IN in 3 chews/day for 3 weeks<br>2) 7.5 g of agave IN in 3 chews/day for 3 weeks	3 chocolate chews/day without IN (fiber content n.d.) for 3 weeks	SCFAs and BCFAs by GC in fecal samples	16S rRNA amplicon sequencing (V4 hyper-variable region)	No significant changes in SCFAs and BCFAs with IN vs. CTRL<br>Positive association between total fiber intake and faecal butyrate<br><br>16S rRNA amplicon sequencing<br>↑ Actinobacteria<br>↑ *Bifidobacteriaceae*<br>↑ *Bifidobacterium*<br>↑ B. adolescentis<br>↑ B. breve<br>↑ B. longum<br>↑ B. pseudolongum<br>↓ Desulfovibrio<br>↓ Lachnobacterium

							↓ Ruminococcus<br><br>Other:<br>Positive correlation between *Bifidobacterium* and agave IN<br>Negative correlation between *Desulfovibrio* and total fiber intake
[13]	20 healthy adult men<br>Age: 27.5 ± 4.3 years<br>BMI: 27.0 ± 4.0 kg/m^2	Randomized, placebo-controlled, double-blind, cross-over trial<br><br>No washout	1) 21 g/day of PDX in 3 snack bars for 3 weeks<br>2) 21 g/day of SCF in 3 snack bars for 3 weeks	Snack bars without supplemental fiber (NFC) (fiber content n.d.) for 3 weeks	SCFAs and BCFAs by GC in fecal samples	16S rRNA amplicon sequencing (V4-V6 hyper-variable regions)	↓ Fecal protein-based fermentative end-products, (ammonia, phenol, indole, and total BCFAs) with PDX, SCF vs. NFC<br>↓ Acetate, propionate, and butyrate with PDX vs. SCF<br>↑ *Clostridiaceae* and *Veillonellaceae* with PDX vs. SCF<br>↓ *Eubacteriaceae* with PDX vs. SCF
[34]	12 healthy adults (4 M, 8 F)<br>Age: 33.9 ± 8.3 years<br>BMI: 23.8 ± 2.4 kg/m^2	Randomized, placebo-controlled, double-blind, cross-over trial<br><br>Washout (3 weeks)	8 g/day of PDX powder for 3 weeks	8 g/day MD powder (fiber content n.d.) for 3 weeks	SCFAs, isovalerate, dimethyl amine, succinate and phenylacetate by NMR in fecal samples	Microbiome data collected in a previous study by Costabile *et al.* 2012 doi: 10.1017/S0007114511005782 (19)	No changes in any fecal metabolites with PDX vs. MD<br>Associations between PDX and acetate and propionate<br><br>A strong correlation between the fecal metabolite profile and the amount of bifidobacteria in feces.<br>*Bacteroides* content had a positive correlation with acetate in feces
[22]	60 healthy adults (26 M, 34 F)<br><br>Placebo group<br>Age: 20.0 ± 0.4* years<br>BMI: 21.6 ± 0.5* kg/m^2<br><br>XOS group<br>Age: 20.1 ± 0.4* years	Randomized, parallel, placebo-controlled, double-blind trial	1) 5 g/day of XOS (from 6.64 g/day of a XOS-enriched compound derived from wheat) for 4 weeks<br>2) 3 g of IN + 1 g XOS (from 6.64 g/day of a mixture containing inulin-type fructans, XOS and MD) for 4 weeks	6.64 g/day of wheat MD (fiber content n.d.) for 4 weeks	SCFAs by GLC in fecal samples	qPCR: Firmicutes spp., *Faecalibacterium prausnitzii*, *Roseburia* spp., and Bacteroidetes spp.	↑ Total SCFAs with INU-XOS vs. MD after 4 weeks<br>↑ Butyrate and propionate with XOS, INU-XOS vs. MD after 4 weeks<br>↓ Acetate with XOS, INU-XOS vs. MD after 4 weeks<br>↑ Propionate:acetate ratio with XOS, INU-XOS vs. MD<br><br>Significant differences in acetate, butyrate and propionate with XOS, INU-XOS vs. baseline<br><br>↑ *Bifidobacterium* with XOS and INU-XOS vs. MD after 4 weeks<br>Moderate ↑ *Lactobacillus* with INU-XOS vs. MD after 4 weeks

	BMI: 20.9 ± 0.6* kg/m²<br><br>INU-XOS<br>Age: 20.1 ± 0.4 years<br>BMI: 21.4 ± 0.5 kg/m²						
[27]	48 healthy adults<br>Age: 28 years<br>BMI: 24.0 ± 3.0 kg/m² (M); 22.0 ± 3.0 kg/m² (F)	Single-center, randomized, parallel, placebo-controlled double-blind, pilot trial	10, 15 or 20 g/day of sugar-free, digestion-resistant dextrin for 2 weeks	20 g/day of GLU (fiber content n.d.) for 2 weeks	SCFAs by GC in fecal samples	RT-qPCR: *Bacteroides fragilis* and *Clostridium perfringens*	No significant changes in SCFAs with NUTRIOSE® vs. baseline<br>↑ Acetate, propionate and butyrate (not significant) after NUTRIOSE® supplementation<br><br>↑ *Bacteroides* and ↓ *C. perfringens* with NUTRIOSE® vs. baseline in both trials
[38]	48 adults with WGTT >35 h<br><br>Arabinoxylan-Oligosaccharide (AXOS) (6 M, 18 F)<br>Age: 36.1 ± 12.9 years<br>BMI: 24.7 ± 3.1 kg/m²<br><br>Maltodextrin (MD) (6 M, 18 F)<br>Age: 35.7 ± 11.0 years<br>BMI: 24.2 ± 2.3 kg/m²	Randomized, placebo-controlled, parallel, double-blind trial	5 g/day of AXOS powder (71% AXOS dm, 10–14% dm β-glucan) for 12 weeks	15 g/day of placebo (MD) (fiber content n.d.) for 12 weeks	SCFAs, BCFAs, succinate and lactate measured by ion exchange chromatography with conductivity detection in fecal samples	16S rRNA amplicon sequencing (V4 hyper-variable region)	No changes in SCFAs, BCFAs, succinate and lactate with AXOS vs. MD<br>↓ Acetate and butyrate with AXOS vs. baseline (not significant)<br><br>↑ *Bifidobacterium* ↓ alpha-diversity after AXOS<br>Lesser ↑ of *Akkermansia*, *Prevotellaceae* NK3831 group and *Lactobacillus*

[50]	17 healthy adults (11 M, 6 F)<br>Age: 25.9 ± 3.2 years<br>BMI: 22.5 ± 2.1 kg/m^2	Randomized, controlled, cross-over trial<br><br>Washout (1 week)	1) 161.0 g/day of OB kernels (RS+DF: 20,2) for 1 evening<br>2) 190.2 g/day of OB cut 1–2 times (CutOB) (RS+DF: 19.4 g) for 1 evening<br>3) 213.0 g/day of RS HAB (31% RS) (RS+DF: 38.1 g) for 1 evening<br>4) 388.0 g/day HBB (~14%, dry basis, mutant 13) (DF+RS: 81.0 g of total fiber) for 1 evening<br>5) 130.0 g/day of WWB added with a high-RS corn starch (Biomin WWB+RS; Hi-maize 1043) (DF+RS: 11.5 g) for 1 evening<br>6) 181.0 g/day of WWB added with the same amount of RS and with DF from barley (WWB+RS+DF) to match the content in OB (DF+RS: 19.1 g) for 1 evening<br>7) 80.5 g/day of OB bread (1/2 OB) (DF+RS:9.9)g of total fiber) for 1 evening	116.7 g/day WWB (3.9 g: 1.3 g RS + 2.6 g DF) for 1 evening	SCFAs by GC in plasma samples	N.A.	↑ Butyrate with HAB, HBB vs. WWB<br>↑ Butyrate and propionate at 30 min after the standardized breakfast than at time 0<br>Positive correlation between indigestible carbohydrate and plasma butyrate<br>Postprandial glucose response inversely related to plasma butyrate and acetate<br>Plasma butyrate concentration at fasting inversely related to the serum insulin response<br>Plasma butyrate positively related to the concentrations of breath H_2
[23]	32 women with low-iron status<br>Age: N.A. (range 18 – 40 years)<br>BMI: 21.5 ± 2.2 kg/m^2	Randomized, double-blind, controlled, cross-over trial<br><br>Washout (2 weeks)	~20 g/day IN for 4 weeks	20 g/day MD (fiber content n.d.) for 4 weeks	SCFAs, isoacids, formate and lactate by HPLC in fecal samples	qPCR: total bacteria and *Bifidobacterium* spp.	No changes in SCFAs, isoacids and formate with IN vs. baseline, MD<br>↑ Lactate with IN vs. baseline, MD<br>Changes in lactate concentration correlated significantly with changes in propionate concentration with IN vs. MD<br>Changes in acetate concentration correlated significantly with changes in butyrate and formate concentration with IN vs. MD<br><br>↑ Bifidobacteria with IN vs. MD
[10]	50 healthy adults<br><br>TRIAL 1 (10 M, 15 F)<br>Age: 31.2 ±	Randomized, placebo-controlled, double-blind, cross-over trials	TRIAL 1: Isocaloric snack bars with 9 ± 7 g fiber (7 g/day IN) for 4 weeks<br><br>TRIAL 2: Isocaloric snack bars with low-dose fiber (5 ± 3 g; 3 g/day IN)	TRIAL 1: Isocaloric snack bars without IN (0± 0 g of fiber) for 4 weeks	SCFAs by GC in fecal samples	16S rRNA amplicon sequencing (V3-V4 hyper-variable regions)	No changes in SCFAs with PRE vs. CTRL both in TRIAL 1 and 2.<br><br>↑ *Bifidobacterium* in TRIAL 1, less increase in TRIAL 2<br>TRIAL 1: ↑ *Cellulomonas*, *Nesterenkonia* and *Brevibacterium*; ↓ *Lachnospira* and *Oscillospira*

	9.1 years<br>BMI: 24.4 ± 3.1 kg/m[2]<br><br>TRIAL 2 (12 M, 13 F)<br>Age: 30.5 ± 8.6 years<br>BMI: 23.8 ± 3.8 kg/m[2]	Washout (4 weeks)	for 4 weeks	TRIAL 2:<br>Isocaloric snack bars with 1 g of IN (1± 0 g of fiber)<br>for 4 weeks			
[44]	17 healthy adults (6 M, 11 F)<br>Age: N.A. (inclusion criterion 20-50 years)<br>BMI: N.A. (inclusion criterion 19-28 kg/m[2])	Researcher-blinded, randomized, exploratory, controlled, cross-over trial<br><br>Washout (5-7 weeks)	WG diet<br>Total fiber: 32 g/day<br>Cereal fiber: 22 g/day<br>Total cereal intake: 259 g/day<br>Total WG intake: 151 g/day<br>for 2 weeks	RG diet<br>Total fiber: 19 g/day<br>Cereal fiber: 8 g/day<br>Total cereal intake: 271 g/day<br>Total WG intake: 0 g/day<br>for 2 weeks	Metabolic profiles by NMR in fecal waters	N.A.	↑ Acetate and butyrate in fecal waters with WG vs. RG<br>↓ Isovalerate in fecal waters with WG vs. RG<br><br>Women:<br>↓ Succinate in fecal waters with WG vs. RG
[14]	12 healthy male adults<br>Age: N.A. (range 27-49 years)<br>BMI: N.A. (exclusion criterion >32 kg/m[2])	Randomized, double-blind, controlled, cross-over trial<br><br>No washout	Controlled diet with 20 g/day of chicory IN into low-fat vanilla ice cream<br>for 3 weeks	Controlled diet with a control ice cream (corn syrup instead of IN) (fiber content n.d.)<br>for 3 weeks	SCFAs by GC in fecal samples	Culture-based analysis: total anaerobic counts, enterobacteria, lactic acid bacteria, *Bifidobacterium* spp. and *Clostridium* spp.	No changes in SCFAs with IN vs. CTRL<br>↑ Acetate:propionate ratio with IN vs. CTRL<br><br>↑ Total anaerobes<br>↑ *Lactobacillus* species<br>↓ *Enterobacteriaceae* (p=0.067)
[35]	37 healthy adults<br><br>Maltodextrin (MD) group (14 M, 4 F):<br>Age: 23.5 ± 2.9 years	Single center, randomized parallel group, double-blind, controlled trial	A low FODMAP diet supplemented with OF 14 g/day<br>for 1 week	A low FODMAP diet supplemented with MD 7 g twice daily (fiber content n.d.)<br>for 1 week	SCFAs by GC-MS in fecal samples	16S rRNA amplicon sequencing (V3-V4 hyper-variable regions)	No changes in SCFAs with OF vs. baseline, MD<br><br>↑ *Bifidobacterium*<br>↓ *Lachnospiraceae*

	BMI: 23.5 ± 2.9 kg/ m^2<br><br>Oligofructose (OF) group (11 M, 8 F):<br>Age: 26.5 ± 12.2 years<br>BMI: 22.5 ± 2.9 kg/ m^2						Negative association between breath H_2 and the abundance of *Blautia*<br><br>*Lachnospiraceae, Roseburia* positively associated with butyrate and acetate<br>*Anaerostipes* negatively associated with isobutyrate (p=0.09) and valeric acid
[26]	14 healthy adults (8 M, 6 F)<br>Age: 22.9 ± 2.1 years<br>BMI: 22.8 ± 2.3 kg/m^2	Single-blinded, randomized, 4-armed, controlled, cross-over trial<br><br>Washout (2 weeks)	1) 3.3 g of beta-glucans (BG corresponding to 1.65 g of mixed-linkage (1→3) (1→4)-β-d-glucan) for 3 weeks<br>2) 3.3 g of mother-line barley (Barley BG) (, 6% BG) for 3 weeks<br>3) 3.3 g of oat BG Mutant barley (BG) (mutant line lys5.f with 16.5–19.8% BG) for 3 weeks	Non-fiber control glucagel for 3 weeks	SCFAs by GC-MS in fecal samples	N.A.	Males:<br>↑ Formate and total SCFAs with barley vs. oat<br>↑ Gamma-amino-butyrate and palmitoleic acid with BG vs. CTRL<br><br>Females:<br>↓ Enterolactone with oat vs. CTRL<br>↓ Enterolactone with BG vs. CTRL<br>↑ Isobutyrate, isovalerate, 2-methylbutyrate with barley vs. oat
[45]	81 healthy adults<br><br>Refined diet (RG) group (25 M, 15 F)<br>Age: 54.0 ± 0.8 years<br>BMI: 26.0 ± 0.5 kg/m^2<br><br>Whole-grain diet (WG) group (24 M, 17 F)<br>Age: 55.0 ± 0.9 years	Randomized, parallel, controlled trial	WG diet<br>Daily consumption:<br>- 207 ± 39 g WG<br>- 40 ± 5 g fiber<br>- 8 ± 1 g soluble fiber<br>- 31 ± 5 g insoluble fiber<br>for 6 weeks	RG diet<br>Daily consumption:<br>- 0 g WG<br>- 21 ± 3 g fiber (6 ± 1 g soluble fiber; 15 ± 2 g insoluble fiber)<br>for 6 weeks	SCFAs by GC-MS in fecal samples	16S rRNA amplicon sequencing (V4 hyper-variable region)	↑ Acetate and total SCFAs with WG vs. RG<br>Correlation between acetate and 19:0 and total alkylresorcinol<br><br>↓ *Enterobacteriaceae*, ↑ *Lachnospira*, ↑ *Roseburia* (trend)<br><br>Positive association between *Lachnospira* and acetate or butyrate

	BMI: 26.0 ± 0.5 kg/m²						
[46]	70 healthy overweight adults (32 M, 38 F): Age: 51.0 ± 9.4 years BMI: 27.8 ± 1.9 kg/m²	Randomized, parallel, controlled trial	Subjects were instructed to replace all cereal products in their habitual diet with the products provided *ad libitum* in the study.<br><br>Registered intake:<br>WGR diet<br>Total dietary fiber: 34.9 ± 11.9 g<br><br>WGW diet<br>Total dietary fiber: 31.1 ± 10.9 g<br>for 6 weeks	RW diet<br>Registered intake:<br>Total dietary fiber: 22.9 ± 9.2 g<br>for 6 weeks	SCFAs by GC in fecal samples	16S rRNA amplicon sequencing (V3-V4 hyper-variable regions)	No significant changes in total SCFAs, acetate, propionate with WGW, WGR vs. baseline<br>↓ Butyrate with RW vs. WGW, WGR<br>Associations between plasma alkylresorcinols and total SCFAs, butyrate and acetate<br><br>No changes in the gut microbiota
[42]	40 healthy adults (22 M, 22 F) Age: 31.4 ± 8.9 years BMI: 23.3 ± 2.8 kg/m²	Randomized, placebo-controlled, double-blind, cross-over trial<br><br>Washout (3 weeks)	7 slices of bread with *in situ* produced AXOS (2.2 g AXOS + 2.0% AX dm for 3 weeks	7 slices of bread<br><br>PLACEBO:<br>Non-endoxylanase-treated breads<br><br>CONTROL:<br>Non-endoxylanase-treated bread<br>for 3 weeks	VFAs by GC in fecal samples	FISH: total bacteria, *Atopobium/ Coriobacterium*, *Bacteroides/ Prevotella*, *Bifidobacterium* spp., *Clostridium histolyticum/perfringens*, *Eubacterium rectale/Clostridium coccoides*, *Lactobacillus/ Enterococcus*, *Escherichia coli*, *Roseburia*, *Faecalibacterium prausnitz*ii	↑ Butyrate with AXOS vs. baseline, pre-treatment<br>↑ Propionate with post-AXOS vs. pre-AXOS<br>↑ Valerate with AXOS, PLA, CTRL breads vs. baseline<br>↓ Iso-valerate with AXOS (*p*=0.058) vs. baseline<br>↑ Combined acetate, propionate and butyrate with AXOS vs. pre-AXOS (*p*=0.072)<br><br>↑ Lactobacilli following PLA and AXOS vs. pre-treatment, then ↓ along post-AXOS treatment washout<br>↑ *Bacteroides and E. rectale* group following PLA and AXOS vs. pre-treatment, and along post-AXOS treatment washout<br>↑ Total bacteria following AXOS treatment
[32]	24 healthy adults (8 M, 16 F) Age: 38.2 ± 7.8 years BMI: 23.1 ± 2.6 kg/m²	Randomized, double-blind, controlled, cross-over trial	21.6 g/day of GOS (containing 15.0 g/day GOS)<br>for 4 weeks	21.6 g/day of MD (fiber content n.d.)<br>for 4 weeks	SCFAs, succinate and isobutyrate by HPLC in fecal samples	qPCR: total bacteria and *Bifidobacterium* spp.<br>16S rRNA amplicon sequencing (V4	No significant changes in SCFAs, isobutyrate and succinate with GOS vs. baseline, MD<br><br>qPCR<br>↑ *Bifidobacterium* spp. after 1 and 4 weeks of GOS supplementation

		Washout (4-6 weeks)				hyper-variable region)	16S rRNA amplicon sequencing<br>- α-diversity:<br>↓ InvSimpson, after 4 weeks of GOS supplementation<br><br>- β-diversity: microbiota composition was significantly different after 1 and 4 weeks of GOS supplementation according to weighted UniFrac<br><br>- Taxa: ↑ *Bifidobacterium* spp. after 1 and 4 weeks of GOS supplementation
[36]	19 healthy adults<br>Age: 46.9 ± 19.7 years<br>BMI: 24.4 (SD N.A.) kg/m²	Randomized, double-blind, controlled, cross-over trial<br><br>Washout (2 weeks)	69 % of OF<br>Week 1: 15 g/day<br>Week 2: 30 g/day<br>for 2 weeks<br><br>83.4 % of AXOS<br>Week 1: 15 g/day<br>Week 2: 30 g/day<br>for 2 weeks	Placebo<br>Week 1: 15 g/day<br>Week 2: 30 g/day<br>for 2 weeks	SCFAs, BCFAs, dimethylsulfide and p-cresol by GC-MS in fecal samples	N.A.	No changes in total and individuals SCFAs, total BCFAs with WBE, OF vs. baseline, PLA<br>↓ Isovalerate with WBE, OF vs. PLA<br>↓ Isovalerate with WBE vs. pre-WBE
[30]	19 adults (9 M, 10 F)<br>Age: N.A. (inclusion criterion 18-55 years)<br><br>Control starch (CS) group<br>BMI: 21.1 ± 0.4 kg/m²<br><br>RS2 group<br>BMI: 21.1 ± 0.4 kg/m²	Randomized, placebo-controlled, double-blind, cross-over trial<br><br>Washout (4 weeks)	40 g of high-amylose RS2 for 4 weeks	Energy-matched CS (fiber content n.d.)<br>for 4 weeks	SCFAs and BCFAs by GC in serum samples	16S rRNA amplicon sequencing (V3 hyper-variable region)	↑ Acetate with RS vs. CS<br>Changes in acetate positively correlated with postprandial change in GLP-1 at 30 min<br><br>↓ *Anaerostipes, Bacteroides, Blautia, Holdemanella, Coprococcus_1, Coprococcus_3, Lachnoclostridium, Lachnospiraceae_UCG-004, Erysipelotrichaceae_UCG-003, Paraprevotella, Phascolarctobacterium, Ruminiclostridium_6, Ruminococcaceae_UCG-002*, and *Eubacterium_eligens* with RS vs. baseline<br>↑ *Ruminococcaceae_UCG-005* with RS vs. CS

[a] N.A.: not available

[b] Interventions: as-is: adjusted for moisture content; AX: arabinoxylan; AXOS: arabinoxylan-oligosaccharides; BB: barley kernel-based bread; ; BG: β-glucans; CF: citrus fiber; CTRL: control; DF: dietary fiber; dm: dry matter; dwb: dry weight basis; FB: fiber blend; FF: fiber free; FODMAP: fermentable, oligo-, di-, mono-saccharides and polyols; FOS: fructooligosaccharide; GLU: glucose; GOS: galacto-oligosaccharide; HAB: high-amylose barley kernels; HBB: high *β*-glucan barley kernels; IN: inulin; LF: lupin kernel fiber; LNnT: lacto-*N*-neotetraose; L-Rha: L-rhamnose; MD: maltodextrin; N.D.: no date; NFC: non-fiber control; NSP: non-starch polysaccharides; NWG: non-whole grain; OB: ordinary barley; OF: oligofructose; PDX: polydextrose; PE-WB: de-starched pericarp-enriched wheat bran; PLA: placebo; PRE: prebiotic; RG: refined-grain; RS: resistant starch; RS2: resistant starch type 2; RS4: resistant starch type 4; RW: refined wheat; SCF: soluble corn fiber; VLCI: very-long-chain inulin; WBE: wheat bran extract; WB-RPS: wheat bran with a reduced particle size; WG: whole-grain; WGM: maize-derived whole-grain cereal; WGO: whole-grain oat Granola; WGR: wholegrain rye; WGW: wholegrain wheat; ww: wet weight; WWB: white wheat bread; XOS: arabinoxylans; 2′FL: 2′-*O*-fucosyllactose

[c] BCFAs: branched-chain fatty acids; ELISA: enzyme-linked immunosorbent assay; FISH: fluorescence *in situ* hybridization; GC: gas chromatography; GC-FID: gas chromatography-flame ionization detector; GC-MS: gas chromatography-mass spectrometry; GLC: capillary gas–liquid chromatography; GM-CSF: granulocyte-macrophage colony-stimulating factor; HPLC: high-performance liquid chromatography; LC-MS/MS: liquid chromatography-tandem mass spectrometry; NMDS: non-metric multidimensional scaling; NMR: nuclear magnetic resonance; OTU: operational taxonomic unit; PCA: principal component analysis; PCR: polymerase chain reaction; qPCR: quantitative polymerase chain reaction; RT-qPCR: real time quantitative PCR; T-RFLP: terminal restriction fragment-length polymorphism; SCFAs: short-chain fatty acids (acetate, propionate, butyrate); UPLC-MS/MS: ultra-performance liquid chromatography - tandem mass spectrometry; VFAs: volatile fatty acids (acetate, propionate, butyrate, caproate, valerate, isobutyrate, isovalerate)

↑: increase; ↓: decrease

Where not specified, SCFAs correspond to acetate, propionate, and butyrate.
Where not specified, total SCFAs correspond to the sum of acetate, propionate, and butyrate.
Where not specified, isoacids correspond to isobutyrate and isovalerate.
Where not specified, BCFAs correspond to valerate, isovalerate, and isobutyrate.
Where not specified, VFAs correspond to acetate, propionate, butyrate, caproate, valerate, isobutyrate, and isovalerate.
*sem value
