# Supplementary material for: Effects of Dietary Fibers on Short-Chain Fatty Acids and Gut Microbiota Composition in Healthy Adults: A Systematic Review
Source: Nutrients. 2022 Jun 21;14(13):2559. doi: 10.3390/nu14132559 (PMC9268559; doi:10.3390/nu14132559)
Supplement: Supplementary file 1 [file nutrients-14-02559-s001.zip › Suppl_Table S2.pdf]

Table S2. Daily fiber intake of participants during the DF interventions reported by studies included in the review.

Reference	Fiber content in the test product[a]	Total daily fiber intake (intervention arm) (g/day)	Fiber content in the control/placebo product	Total daily fiber intake (control/placebo arm) (g/day)
**[28] Alfa**	30 g MSPrebiotic® RS (70% RS2; from test product)	N.D.	N.D.	N.D.
**[43] Ampatzoglou**	N.D.	26 ± 1	N.D.	16 ± 1
**[11] Boler**	21 g PDX (from 3 snack bars g/day)<br><br>21 g SCF (from snack bars g/day)	PDX: 14.8 (supplemental fiber is not included)<br><br>SCF: 14.4 (supplemental fiber is not included)	Control bar total fiber 0.9 %	14.8
**[31] Boll**	8.9 g AXOS ww + 1.5 g Glucans ww + 6.6 g RS ww (from a 140.5 g portion sizes of the test products)<br><br>18.4 g AXOS ww + 3.1 g glucans ww + 1.0 g RS ww (from a 141.4 g portion sizes of the test products)<br><br><0.15 g Glucans ww + 15 g RS ww (from a 137.9 g portion sizes of the test products)	N.D.	<0.15 g Glucans ww + 1.2 g RS ww (from a 116.2 g portion sizes of the test products)	N.D.
**[9] Brandl**	TRIAL 1<br>10 g extrinsic wheat fiber (from experimental solid foods)<br><br>TRIAL 2<br>10 g extrinsic wheat fiber (wheat plant fiber 3.75%, psyllium 3.75%, guar 3.75%, from powder dissolved in 300 mL water)	TRIAL 1: 35.0 ± 6.0<br><br>TRIAL 2: 35.0 ± 10.0	TRIAL 1: N.D.<br><br>TRIAL 2: placebo powdered cellulose 3.75%; psyllium 3.75%; guar 3.75%	TRIAL 1: 22.0 ± 7.0<br><br>TRIAL 2: 25.0 ± 10.0
**[47] Carvalho-Wells**	14.2 g total fiber (from a 48 g/day serving of product)	N.D.	0.81 g total fiber (from a 48 g/day serving of product)	N.D.

**[24] Chen**	15 g arabinogalactan (from test product)	14.41 ± 6.10 (supplemental fiber is not included)	N.D.	15.01 ± 7.24
**[15] Clarke**	3×5 g/day β_2-1 fructan (50:50 mixture of IN and short-chain oligosaccharides; from a test product)	N.D.	N.D.	N.D.
**[48] Connolly**	~ 2.8 g fiber and 1.3 g -glucan (from a 45 g/day serving of product)	18.8 ± 0.9	~1.35 g fiber (from a 45 g/day serving of product)	18.2 ± 1.1
**[16] Costabile 2010**	10 g/day VLCI (inulin: 97.5 %; from a test product)	N.D.	N.D.	N.D.
**[33] Costabile 2012**	8 g/day PDX powder (from a test product)	N.D.	N.D.	N.D.
**[41] Damen**	4.0% AX dm + 2.14 g AXOS + 9.2 % total fiber (from 180 g/day serving of product)	N.D.	3.7% AX dm + 10.1 % total fiber (from 180 g/day serving of product)	N.D.
**[17] Darzi**	25.5 g/day L-Rha (from test products)<br><br>22.4 g/day IN (from test products)	N.D.<br><br>N.D.	N.D.	N.D.
**[29] Deehan**	65.0/58.2 dwb/as-is % total fiber (from test product of maize RS4)<br><br>90.0/78.7 dwb/as-is % total fiber (from test product of potato RS4)<br><br>96.0/85.3 dwb/as-is % total fiber (from test product of tapioca RS4)	N.D.	0.0	N.D.
**[18] Deroover**	50 % fiber (from WB fraction – unmodified WB) + 10 g ^{13}C-inulin + 10 g inulin-^{14}C-carboxylic acid (from a standard breakfast)<br><br>50 % fiber (from 20 g wheat bran with a reduced particle size) + 10 g ^{13}C-inulin + 10 g inulin-^{14}C-carboxylic acid (from a standard breakfast)	N.D.	10 g ^{13}C-inulin + 10 g inulin-^{14}C-carboxylic acid (from a standard breakfast)	N.D.

	71 % fiber (from 20 g de-starched pericarp-enriched wheat bran) + 10 g ^{13}C-inulin + 10 g inulin-^{14}C-carboxylic acid (from a standard breakfast)			
**[49] Duysburgh**	1.4 g β-glucans (from a 40 g/day serving of product)	N.D.	0 (from a 40 g/day serving of product)	N.D.
**[39] Elison**	5, 10 or 20 g/day 2′FL (from a test product)<br><br>5, 10 or 20 g/day LNnT (from a test product)<br><br>5, 10 or 20 g/day 2′FL+LNnT (2:1 mass ratio; from a test product)	N.D.	N.D.	N.D.
**[19] Fernandes**	24 g IN (from 300 ml drink)	N.D.	N.D.	N.D.
**[12] Fernando**	N.D. (expected to contain 3-5 g/day of oligosaccharides)<br><br>5 g/day of raffinose (from test products)	N.D.	N.D.	N.D.
**[40] Fechner**	25 g/day lupin kernel fiber (from experimental foods)<br><br>25 g/day citrus fiber (from experimental foods)	59.1 ± 5.6<br><br>57.8 ± 5.6	N.D.	34.4 ± 5.8
**[25] Finegold**	175 mg XOS (from 1.4 g/day capsule supplements) for 8 weeks<br><br>350 mg XOS (from 2.8 g/day capsule supplements)	N.D.<br><br>N.D.	N.D.	N.D.
**[37] François**	2.4 g AXOS (from 140 ml soft drinks with WBE at 3 g/day)<br><br>8 g AXOS (from 140 ml soft drinks with WBE at 10 g/day)	N.D.<br><br>N.D.	N.D.	N.D.
**[20] Healey**	16 g/day of powdered inulin-type fructan prebiotic (50:50 inulin to fructo-oligosaccharide mix)	28.2 ± 12.2	N.D.	28 ± 11.9
**[21] Holscher**	5.0 g agave IN (from 3 chews/day)<br><br>7.5 g agave IN (from 3 chews/day)	17.6 (without agave IN)<br><br>18.0 (without agave IN)	N.D.	18.2

**[13] Hooda**	21 g PDX (from 3 snack bars/day)<br><br>21 g of SCF (from 3 snack bars/day)	PDX: 14.8 (supplemental fiber is not included)<br><br>SFC: 14.4 (supplemental fiber is not included)	Control bar total fiber 0.90%	14.8
**[34] Lamichhane**	8 g/d PDX powder (from a test product)	N.D.	N.D.	N.D.
**[22] Lecerf**	5 g XOS (from 6.64 g/day of a XOS-enriched compound derived from wheat)<br><br>3 g IN + 1 g XOS (from 6.64 g/day of a mixture containing inulin-type fructans, XOS and MD)	12.1 ± 0.6 (without XOS)<br><br>N.D. (INU-XOS group)	N.D.	N.D.
**[27] Lefranc-Millot**	NUTRIOSE® (sugar-free, digestion-resistant dextrin) 10, 15 or 20 g/day	N.D.	N.D.	N.D.
**[38] Müller**	71% AXOS dm, 10–14% dm β-glucan (from a 5 g/day of AXOS powder)	18.3 ± 6.9	N.D.	18.3 ± 9.4
**[50] Nilsson**	20.2 g (9.5 RS + 10.7 DF; from 161.0 g/day serving of product)<br><br>19.4 g (8.8 g RS + 10.6 g DF; from 190.2 g/day serving of product)<br><br>38.1 g (22.0 g RS + 16.1 g DF; from 213.0 g/day serving of product)<br><br>81.0 g (30.9 g RS + 50.1 g DF; from 388.2 g/day serving of product)<br><br>11.5 g (8.0 g RS + 3.5 g DF; from 130 g/day serving of product)<br><br>19.1 g (8.8 g RS + 10.3 g DF; from 181.8 g/day serving of product)	N.D.	3.9 g (1.3 g RS + 2.6 g DF; from 116.7 g/day serving of product)	N.D.

	9.9 g (4.7 g RS + 5.2 g DF; from 80.5 g/day serving of product)			
**[23] Petry**	~20 g/day IN (from test products)	N.D.	N.D.	N.D.
**[10] Reimer**	TRIAL 1: 9 ± 7 g (from 40 g/day serving of product)<br><br>TRIAL 2: 5 ± 3 g (from 25 g/day serving of product)	N.D.<br><br>N.D.	TRIAL 1: 0 ± 0 (from 37 g/day serving of product)<br><br>TRIAL 2: 1 ± 0 (from 28 g/day serving of product)	N.D.<br><br>N.D.
**[44] Ross**	N.D.	Females: 30 ± 2<br><br>Males: 34 ± 3.6	N.D.	Females: 18 ± 1<br><br>Males: 20 ± 1.6
**[14] Slavin**	20 g chicory IN (from low-fat vanilla ice cream)	N.D.	N.D.	N.D.
**[35] Sloan**	14 g/day OF	19.7 ± 5.8 (supplemental fiber is not included)	N.D.	19.6 ± 7.6
**[26] Trimigno**	3.3 g β-glucan (oat BG)<br><br>3.3 g β-glucan (barley BG)<br><br>3.3 g β-glucan (barley mutant BG)	23.2 ± 2.7<br><br>28.7 ± 4.2<br><br>24.2 ± 2.4	Non-fiber control	26.6 ± 2.8
**[45] Vanegas**	N.D.	40 ± 5 during follow-up (week 3 to week 8)	N.D.	21 ± 3 during follow-up (week 3 to week 8)
**[46] Vuholm**	<u>WGR diet</u><br>12.4 g/100 g of DF of rye flakes<br>10.6 g/100 g of DF of rye bun (light)<br>11.7 g/100 g of DF of rye bun (dark)<br>8.3 g/100 g of DF of rye WG pasta<br>12.5 g/100 g of DF of rye kernels<br>12.0 g/100 g of DF of WG crisp bread<br>Amount of study products consumed (g/day): 217±20.2<br><br><u>WGW diet</u><br>9.5 g/100 g of DF of wheat flakes<br>8.0 g/100 g of DF of wheat bun (light)	<u>WGR diet</u><br>34.9 ± 11.9<br><br><u>WGW diet</u><br>31.1 ± 10.9	<u>RW diet</u><br>3.7 g/100 g DF of puffed wheat<br>5.1 g/100 g DF of Sandwich bread<br>4.0 g/100 gDF of rustic rolls<br>3.0 g/100 g DF of refined pasta<br>5.7 g/100 g DF of pearled spelt kernels	22.9 ± 9.2

	10.1 g/100 g of DF of wheat bun (dark)<br>8.3 g/100 g of DF of wheat WG pasta<br>9.6 g/100 g of DF of WG wheat kernels<br>7.9 g/100 g of DF of WG crisp bread<br>Amount of study products consumed (g/day): 239±21.2		4.8 g/100 g DF of crisp bread<br>Amount of study products consumed (g/day): 221±22.9	
**[42] Walton**	2.0% AX dm + 2.2 g AXOS/day (from a 180 g/day serving of product)	18.9 ± 5.7	control AX content 0.6% dm<br>placebo 0.9% dm<br>4.2% total AX	19.8 ± 7.9
**[32] Wilms**	15.0 g/day of pure GOS (from 21.6 g/day of Biotis™ GOS)	19.5 ± 6.6 (supplemental fiber not included)	N.D.	19.4 ± 7.2
**[36] Windey**	83.4 % of dm of AXOS (from 15 g/day and 30 g/day of test product 1)<br>or<br>69 % of OF (from 15 g/day and 30 g/day of test product 2)	N.D.	N.D.	N.D.
**[30] Zhang**	40 g/day high-amylose RS2	54.99 ± 6.62	N.D.	10.04 ± 0.57

[a] Interventions: as-is: adjusted for moisture content; AX: arabinoxylan; AXOS: arabinoxylan-oligosaccharides; BG: β-glucans; DF: dietary fiebr; dm: dry matter; dwb: dry weight basis; IN: inulin; LNnT: lacto-N-neotetraose; L-Rha: L-rhamnose; N.D.: no date; OF: oligofructose; PDX: polydextrose; RS: resistant starch; RS2: resistant starch type 2; RS4: resistant starch type 4; RW: refined wheat; SCF: soluble corn fiber; VLCI: very-long-chain inulin; WB: wheat bran; WBE: wheat bran extract; WGR: wholegrain rye; WGW: wholegrain wheat; ww: wet weight; XOS: xylo-oligosaccharide; 2′FL: 2′-O-fucosyllactose
