# Supplementary material for: Effects of Dietary Fibers on Short-Chain Fatty Acids and Gut Microbiota Composition in Healthy Adults: A Systematic Review
Source: Nutrients. 2022 Jun 21;14(13):2559. doi: 10.3390/nu14132559 (PMC9268559; doi:10.3390/nu14132559)
Supplement: Supplementary file 1 [file nutrients-14-02559-s001.zip › Suppl_Table S3.pdf]

Table S3. Characteristics of the health-related outcomes and their findings.

Reference	Other Outcomes[d]	Other Findings[d]
[28]	Tolerance, GI symptoms and stool characteristics	No significant changes in overall health, abdominal pain, bloating, flatulence, bowel habits with MSPrebiotic® RS vs. Amioca TF
[43]	Biochemical markers, anthropometric characteristics, PB, glucose response, stool characteristics and GI symptoms	↑ Body weight (*p*=0.10), BMI (*p*=0.008) with RG vs. WG<br>↑ Frequency in diarrhea/loose stool with WG vs. RG<br>Negative correlation between total fiber intake and body fat %<br>Positive correlation between 24-h fecal weight and total fiber intake
[11]	Flatulence, distention, reflux, tolerance scores, fecal ammonia, 4-methylphenol and indole	↑ Flatulence (*p*=0.001) and distention (*p*= 0.07) with PDX, soluble maize fiber vs. NFC<br>↓ Fecal ammonia, 4-methylphenol and indole with PDX, soluble maize fiber vs. NFC<br>↓ Fecal pH with soluble maize fiber vs. NFC<br>↑ Fecal wet weight and reflux with soluble maize fiber vs. NFC
[31]	Glucose, $ISI_{composite}$, NEFA, GLP-1, GLP-2, breath H_2, subjective appetite and GI symptoms	↓ $ISI_{composite}$ with WWB + hiAXOS vs. WWB<br>↓ Dose-dependent in glucose response and fasting insulin with increasing AXOS<br>↑ Dose-dependent in $ISI_{composite}$ with increasing AXOS<br>↑ Breath H_2 with AXOS vs. WWB<br>↑ Dose-dependent in breath H_2 with increased AXOS at fasting and during 3 h
[9]	Anthropometry and body composition, fecal bulk, stool consistency and frequency	No changes in anthropometric and body composition parameters in PRE (trial 1, trial 2) vs. baseline<br><br>TRIAL 1:<br>↑ Fecal wet weight with PRE vs. CTRL
[47]	Blood lipids, glucose, anthropometric measurements and bowel habits	No changes in serum lipids, glucose, bowel habits and anthropometric measures according to treatment or in washout
[24]	Bowel habit, stool consistency, GI tolerance symptoms, chemistry profile, metabolic panel and vitals	↑ Gene abundance of α-L-rhamnosidase, β-fructosidase, and levanase, and tricarboxylic acid and vitamin B6 biosynthesis pathways with arabinogalactan vs. MD
[15]	Wellbeing, gut symptomology, circulating immune cell populations and cytokine profiles and blood biochemistry	↑ GI symptoms with Orafti vs. baseline, MD<br>↑ Indigestion with Orafti vs. baseline, MD<br>↑ Abdominal pain with Orafti vs. baseline, MD<br>↑ Frequency of self-reported adverse GI events with Orafti vs. MD<br>↑ Serum LPS with Orafti vs. baseline, MD<br>↑ % of CD282+/TLR2+ myeloid dendritic cells with Orafti vs. MD<br>↑ T helper 2 IL-4 and GM-CSF with Orafti vs. MD<br>↓ IL-10 with Orafti vs. MD

		Differences in serum and fecal Ig concentrations with Orafti vs. baseline, MD
[48]	TC, HDL, LDL, TAG, glucose, CRP, IL-6, TNF-α, PYY, GLP-1 and insulin concentrations	Significant time x treatment interaction for TC and LDL<br>↓ TC with WGO after 6 wks vs. baseline<br>↑ TC and LDL with NWG vs. baseline
[16]	GI symptoms, bowel habits and stool characteristic	↑ Mild and moderate bloating with VLCI vs. MD<br>Highly variable stool consistency
[33]	Immunological analysis (IgA and PGE_2), DNA damage, bowel habits and GI symptoms	No changes in bowel habits and GI symptoms except for ↑ formed stools with PDX vs. MD<br>↓ Total fecal IgA and genotoxic damage to HT29 DNA with PDX vs. MD
[41]	Urinary phenol and p-cresol excretions, stool frequency and consistency, and adverse GI symptoms	↓ Urinary phenol and p-cresol excretions with WR+ vs. WR-<br>↑ Stool frequency with WR+ vs. WR-
[17]	Postprandial metabolites, insulin sensitivity, glucose, quantitative and qualitative appetite assessment, breath H_2, NEFA and GI symptoms	↑ Fasting AUC breath H_2 with IN vs. L-Rha<br>↑ Breath H_2 with IN, L-Rha vs. CTRL<br>↓ iAUC with L-Rha vs. CTRL<br>Significant treatment x time effects for postprandial insulin concentrations<br>Treatment × time interaction following lunch for NEFA<br>↑ GI symptoms with IN vs. CTRL<br>↑ Urge to defecate with L-Rha vs. CTRL (day 7)
[29]	Gl tolerance and bowel habits	↑ Composite GI scores with maize and tapioca RS4 vs. baseline<br>↑ Bowel movement frequency with potato RS4 at 50 g/d vs. baseline<br>↓ Fecal hardness with potato RS4 at ≥35 g/d vs. baseline
[18]	Start and duration of fermentation	No differences in the start and duration of fermentation with WB fractions vs. CTR
[49]	N.A.	N.A.
[39]	Blood analysis, GI symptoms, safety and tolerance	Supplementation of 20 g of 2′FL and LNnT was safe and well tolerated: no irregularities in blood analysis<br>↑ Bloating and passing of gas with 20 g of 2′FL and LNnT vs. baseline<br>↑ Rumbling with 20 g dose of 2′FL vs. baseline<br>↑ Harder stools with 20 g LNnT vs. baseline
[19]	Glucose, insulin, C-peptide, FFA, breath H_2 and methane concentrations	↑ Breath H_2 and methane responses with IN vs. GLU<br>↑ Breath H_2 and methane AUC with IN vs. GLU<br>↓ FFA rebounded with IN vs. GLU<br>↓ FFA 4 h with IN vs. GLU
[12]	N.A.	N.A.
[40]	Anthropometric characteristics, blood lipids and BP	↓ Body weight, BMI, fat-free mass, waist circumference, with CF, LF vs. baseline<br>↓ Fecal pH, body fat mass, TC, LDL, total:HDL and LDL:HDL, hs-CRP with LF vs. baseline<br>↓ Systolic BP and fecal dry matter with CF, LF vs. CD<br>↓ Blood lipid markers (except for HDL) with LF vs. CD, CF

		↓ TC, HDL-C, LDL-C with CF vs. baseline<br>↑ Daily fecal weight with LF vs. CD<br>↓ OFTT wih LF vs. CD<br>↑ Excretion of primary bile acids with LF vs. baseline, CF<br>↓ Excretion of total bile acids and secondary bile acids with CF vs. CD
[25]	Tolerance of XOS, GI symptoms and stool characteristics	No changes in stool pH, mass and GI side effects with XOS vs. PLA
[37]	Emergent adverse events, hematological and clinical chemistry parameters, tolerance and effects on colonic protein and carbohydrate fermentation	↓ Stool pH, p-cresol and frequency of constipation with WBE at 10 g/day vs. PLA<br>↓ % lymphocytes with WBE at 3 g/day vs. MD<br>↑ Frequency and severity of flatulence with WBE at 10 g/day vs. PLA
[20]	Appetite ratings and GI symptoms	LDF group:<br>No significant changes in appetite rating with Orafti vs. baseline<br><br>HDF group:<br>↑ Frequency of moderate GI symptoms (flatulence) with Orafti vs. MD<br>↓ Satisfaction before lunch and hunger before dinner with Orafti<br>↑ Fullness and satisfaction after lunch with Orafti
[21]	Gl tolerance, daily stool characteristics and daily food intake	7.5 g/day of agave IN was well tolerated<br>↓ Fecal 4-methyphenol and fecal pH (not significant) with IN vs. CTRL
[13]	Fecal protein-based fermentative end-products and fecal pH	↓ Fecal pH with SCF vs. NFC
[34]	Biogenic amine (dimethyl amine), organic acid (succinate), and amino acid (phenylacetate)	No changes in any fecal metabolites
[22]	Fecal dry matter and pH, p-cresol and phenol, α-glucosidase and β-glucuronidase activities, sIgA and tolerance	↑ Digestive tolerance with INU-XOS vs. MD, XOS after 3 wks<br>↑ Flatulence, bloating and daily stool frequency with INU-XOS vs. MD after 3-4 weeks<br>↑ Liquidity perceived of stool with INU-XOS vs. MD after 3 weeks<br>↓ Stool consistency with INU-XOS vs. MD after 3-4 weeks<br>↓ LPS, general wellbeing and professional activities with INU-XOS vs. MD after 4 weeks<br>↓ Fecal pH with XOS vs. MD after 4 weeks<br>↓ p-cresol with XOS vs. MD<br>↑ Bacterial enzyatic activity with XOS, INU-XOS vs. MD after 4 weeks<br>↑ Fecal expression of s-IgA with INU–XOS vs. MD<br>↑ sIgA with INU-XOS vs. MD (not significant)
[27]	β-glucosidase activity, fecal pH and tolerance	↑ β-glucosidase activity and frequency flatulence (but milder) with NUTRIOSE® at 10 and 15 g/day vs. GLU<br>↓ Fecal pH with NUTRIOSE® at 20 g/day vs. baseline<br>↑ Incidence of abdominal pain with GLU vs. NUTRIOSE® at 10 and 15 g/day

[38]	WGTT, stool parameters, gut permeability, plasma LBP, fecal calprotectin, plasma IL-6, IL-8, TNF-α, and IL-1β, energy expenditure, substrate metabolism, GLP-1 and PYY	No changes in WGTT, gastric emptying, OCTT, gut permeability, plasma LBP, fecal calprotectin, plasma IL-6, IL-8, TNF-α, and IL-1β, energy expenditure, respiratory quotient and carbohydrate oxidation, glucose, insulin, FFA, TAG, glycerol and appetite, hunger, satiety, and fullness ratings with AXOS vs. MD<br>No changes in stool frequency, stool weight, stool moisture and fasting H_2 with AXOS vs. baseline<br>↑ Bristol stool scale with AXOS vs. baseline<br>↓ Postprandial GLP-1 $AUC_{0\text{-}90min}$ with AXOS vs. MD
[50]	Relationship between SCFAs and metabolic test markers	
[23]	Iron status (hemoglobin, PF, and CRP)	↑ iron absorption with IN vs. MD (not significant)<br>↓ Fecal pH with IN vs. baseline, MD
[10]	GI symptoms, body weight and quality of life	No changes in body weight within or between PRE vs. CTRL in both trials and in GI symptoms with PRE vs. CTRL in both trials
[44]	Metabolic profiles of plasma, urine, and fecal waters	After 1 week:<br>↑ Nicotinurate in fecal waters with WG vs. RG<br>↓ Urinary carnitine, acetylcarnitine, urea and taurine with WG vs. RG<br><br>After 2 weeks:<br>↑ Plasma urea with WG vs. RG<br><br>Men:<br>↓ 4-hydroxyphenylacetate, dimethylamine, trimethylamine and methylguanadine, pyruvate, citrate, succinate, 3-hydroxyisovalerate and N-acetyl-glycoproteins with WG vs. RG<br>↑ creatinine with WG vs. RG<br><br>Women:<br>↑ fumarate at week 1 with WG vs. RG
[14]	Stool weight, intestinal transit time, stool frequency and consistency, selected intestinal enzymes, fecal pH and ammonia	↓ Ammonia levels and β-glucuronidase activity with IN vs. CTRL<br>↑ Flatulence with IN vs. CTRL
[35]	Urine metabolites, colonic volume	↑ Colonic volume with OF, MD vs. baseline<br>↑ Fasting breath H_2 with OF vs. MD<br>↓ Aggregate metabolite score for carbohydrates and carbohydrate conjugates with OF, MS vs. baseline<br>No changes in aggregate metabolite scores for amino acids, peptides and analogues and lipids with OF vs. baseline
[26]	Total cholesterol differences	

[45]	Salivary/stool IgA and stool/plasma cytokines, stool characteristics, blood lipid profile, DTH, differential white blood cell count, lymphocyte phenotype, and lymphocyte proliferation,<br>plasma cytokines and LBP, *ex vivo* production of cytokines and NK cell activity	↑ Total effector memory with WG vs. RG<br>↑ LPS-stimulated TNF-α production with WG vs. RG<br>↑ Stool weight and stool frequency with WG vs. RG
[46]	Flatulence, stool frequency, fecal pH, GI symptoms, intestinal permeability and breath H_2	↑ Flatulence with WGW, WGR vs. RW<br>↓ Bloating with WGW, WGR vs. RW<br>↑ Stool frequency with WGR vs. RW at weeks 2 and weeks 4<br>↑ Soft and water content in stool with WGR vs. baseline
[42]	Salivary sIgA assessment, bowel habits and general mood	No changes in salivary sIgA levels, bowel habits and general mood with AXOS vs. pre-AXOS
[32]	Volatile organic compounds, immune parameters, TEAC, GI tolerance and stool characteristics	No changes in breath metabolites, immune markers, GI tolerance, stool characteristics and parameters of systemic oxidative stress with GOS vs. MD
[36]	Fecal water genotoxicity and cytotoxicity	↓ Fecal p-cresol and water cytotoxicity with WBE vs. pre-WBE<br>↓ Fecal p-cresol and water cytotoxicity with WBE, OF vs. PLA<br>Significant negative correlation between fecal output and cytotoxicity
[30]	Safety, GI symptoms, body fat, anthropometric and biochemical assessments, metabolomics profiling of serum and urine, insulin secretion, liver function indices (ALT, AST, GGT), glucose metabolism and gut hormones	No gastrointestinal adverse events reported; safety of RS supplementation confirmed by clinical chemistry and metabolomics analyses<br>↓ Abdominal adiposity, LDL-cholesterol, UA and blood urea nitrogen with RS vs. CS<br>↑ Insulin, C-peptide and active GLP-1 at 30 min after the meal with RS vs. CS<br>↑ AUC for C-peptide with RS vs. CS

[d] ALT: alanine aminotransferase; AST: aspartate aminotransferase; AUC: area under curve; BP: blood pressure; CRP: C-reactive protein; DTH: delayed-type hypersensitivity; FFA: free fatty acids; GGT: γ-glutamyl transferase; GI: gastrointestinal; GLP: glucagon-like peptide; HDL: HDL-cholesterol; HOMA: homeostasis assessment model; hs-CRP: high-sensitivity C-reactive protein; H_2: hydrogen; iAUC: incremental area under curve; IgA: immunoglobulin A; $ISI_{composite}$: insulin sensitivity index; LBP: LPS-binding protein; LDL: LDL-cholesterol; LPS: lipopolysaccharide; NEFA: non-esterified fatty acids; NK: natural killer; OCTT: oro-cecal transit time; OFTT: oro-fecal transit time; OXM: oxyntomodulin; PF: plasma ferritin; PGE_2: prostaglandin E_2; PYY: peptide YY; sIgA: secretory immunoglobulin A; TAG: triacylglycerol; tAUC: total areas under the curve; TC: fasting total cholesterol; TEAC: trolox equivalent antioxidant capacity; UA: uric acid; VLDL: very-low density lipoproteins; WGTT: whole-gut transit time

↑ : increase; ↓ : decrease
